# Exosomal *miR-375-3p* mediated lipid metabolism, ferritinophagy and CoQ-dependent pathway contributes to the ferroptosis of keratinocyte in SJS/TEN

**DOI:** 10.7150/ijbs.98592

**Published:** 2025-01-20

**Authors:** Chen Zhang, Pei Qiao, ChunYing Xiao, ZiPeng Cao, JiaoLing Chen, Hui Fang, JianKang Yang, ZeHua Kang, ErLe Dang, Shuai Shao, BingYu Pang, QingYang Li, ZhenLai Zhu, ShengXian Shen, Akito Hasegawa, Riichiro Abe, HongJiang Qiao, Gang Wang, Meng Fu

**Affiliations:** 1 Department of Dermatology, Xijing Hospital, Fourth Military Medical University, Xi'an, 710032, China.; 2 Department of Health Education and Management and the Ministry of Education Key Lab of Hazard Assessment and Control in Special Operational Environment, School of Public Health, Fourth Military Medical University, Xi'an, 710032, China.; 3 Division of Dermatology, Niigata University Graduate School of Medical and Dental Sciences, Niigata, 951-8510, Japan.

**Keywords:** Exosomes, miRNA, ferroptosis, ferritinophagy, lipid metabolism, Coenzyme Q10, Stevens-Johnson syndrome, toxic epidermal necrolysis

## Abstract

Stevens-Johnson syndrome (SJS) and toxic epidermal necrolysis (TEN) manifest life-threatening cutaneous adverse drug reactions characterized by keratinocyte death. Previous studies have indicated that apoptosis and necroptosis are implicated in SJS/TEN pathogeneses. However, other forms of cell death involved in this process remain unidentified. Ferroptosis, cell death driven by iron-dependent lipid peroxidation, has been implicated in various human diseases. In this study, the identification of ferroptosis and the potential effects of ferroptosis on SJS/TEN were investigated. We demonstrated that the skin lesions and plasma of SJS/TEN patients show increased levels of lipid peroxidation and iron. The biomarkers of ferroptosis correlated positively with the disease severity in SJS/TEN patients. Besides, plasma exosomes derived from patients with SJS/TEN exhibited elevated levels of cellular oxidized polyunsaturated fatty acids (PUFAs) and phospholipids phosphatidylethanolamine (PE), the key phospholipids that drive cells towards ferroptotic death. *miR-375-3p*, enriched in plasma-derived exosomes from SJS/TEN patients, was observed reduce both ferritin heavy chain 1 (FTH1) and ferroptosis suppressor protein 1 (FSP1) expression. Parallelly, exosomal *miR-375-3p* overexpression increased the level of lipid peroxidation but decreased the coenzyme Q10 (CoQ10), thus enhancing the ferroptosis rate of keratinocyte. Above all, we concluded that ferritinophagy-mediated ferroptosis, lipid metabolism, and the FSP1-CoQ-dependent pathway in ferroptosis are critical mechanisms contributing to SJS/TEN. Targeting ferroptosis in keratinocyte may be a therapeutic strategy for preventing SJS/TEN in the future.

## Introduction

SJS and TEN are severe, life-threatening mucocutaneous reactions characterized by widespread keratinocyte death, blisters, and mucosal sloughing. Drugs are the main causes of these diseases 1, 2. SJS/TEN can lead to complications in the liver, kidneys, and respiratory tract 2. Histologically, the skin of SJS/TEN patients separates at the dermo-epidermal junction accompanied by apoptosis and necrosis of epidermal keratinocyte. Classical apoptosis and necroptosis are the relevant cell death pathways critical for the extensive keratinocyte death observed in SJS/TEN 3, 4. However, other forms of cell death involved in this process remains to be elucidated.

Recently, another programmed necrosis modality known as ferroptosis has been described. Ferroptosis is a novel mode of non-apoptotic cell death induced by the build-up of toxic lipid peroxides in an iron dependent manner 5, 6. Among the various lipids, PUFAs associated with PE, is responsible for ferroptosis-inducing lipid peroxidation 7. Lipidomic studies suggest that PEs containing arachidonic acid (C20:4), adrenic acid (C22:4) or docosahexanoic acid (C22:6), are key phospholipids that drive cells towards ferroptotic death 8. Acyl-CoA synthetase long-chain family member 4 (ACSL4), lysophosphatidylcholine acyltransferase 3 (LPCAT3), and arachidonate 15-lipoxygenase (ALOX15), are critical enzymes involved in the incorporation of PUFAs into phospholipids in ferroptosis 7. In addition, FSP1 has been identified as a potent ferroptosis resistance factor, and the FSP1-CoQ10-reduced nicotinamide adenine dinucleotide (phosphate) [NAD(P)H] pathway, which has been identified as a strong inhibitor of lipid peroxidation and ferroptosis 9, 10. Besides, nuclear receptor coactivator 4 (NCOA4) is a selective cargo receptor that mediates the autophagic degradation of ferritin, in a process known as ferritinophagy 11. Specifically, NCOA4 selectively interacts with the FTH1 subunit of ferritin 12. FTH1 plays an important role in the maintenance of the cellular iron balance in ferroptosis.

Ferroptosis has been recently associated with severe pathological conditions, including carcinogenesis, stroke, kidney ischemia/reperfusion (I/R) injury, and degenerative diseases 6. For these conditions, ferroptosis prevention is an attractive therapeutic option. However, whether ferroptosis occurs in SJS/TEN and whether it plays an important role in their pathogenesis remains unknown.

Exosomes, small extracellular vesicles, carry diverse bioactive molecules capable of facilitating inter-organ communication 13. Many microRNAs (miRNAs) are found in secreted exosomes and are extensively involved in many apoptotic and non-apoptotic processes 14-18. We previously demonstrated that exosomal *miR-375-3p* promotes keratinocyte apoptosis via *X-linked inhibitor of apoptosis (XIAP)* repression in SJS/TEN 19. Interestingly, *miR-375-3p* can also induce ferroptosis by targeting solute carrier family 7 member 11 (*SLC7A11*), a negative regulatory factor of ferroptosis, and thus attenuate the stemness of gastric cancer cells and cervical cancer progression 20-22. However, whether exosomal *miR-375-3p* is involved in keratinocyte ferroptosis in SJS/TEN is largely unknown.

Therefore, we aimed to determine whether ferroptosis is involved in SJS/TEN pathogenesis and its potential effects. In this study, we demonstrated exosomal *miR-375-3p* promoting cellular oxidized PUFA, substantially downregulating both FSP1 and FTH1 expression, and inducing the CoQ-dependent and ferritinophagy-mediated ferroptosis pathway in SJS/TEN. Our study implied that targeting ferroptosis in keratinocyte may be a promising strategy for preventing SJS/TEN in the future.

## Materials and methods

### Subjects and sample collection

Plasma from 17 SJS patients (4 males and 13 females; average age, 31.24 y) and 18 TEN patients (9 males and 9 females; average age, 40.89 y) were obtained from the Department of Dermatology in the Xijing Hospital of the Fourth Military Medical University, Shaanxi, China. The clinical characteristics of the SJS/TEN patients are listed in **Table S1**. This study included the blood samples of patients with SJS/TEN, erythema multiforme (EM), and healthy individuals. Skin lesions were excised from the skin of 10 patients with SJS/TEN, EM, or maculopapular drug eruption (MPE), which had been diagnosed clinically and pathologically. Control skin samples were obtained from age-and sex-matched healthy individuals undergoing plastic surgery. Institutional review board approval (IRB number: KY20172030-1) was obtained before patients and healthy volunteers were enrolled in the study in accordance with the Declaration of Helsinki. The detailed contents of the institutional review board ethical approval are shown in **Table S2**.

### Cell culture and treatment

Primary human keratinocytes were isolated from human juvenile foreskin and cultured in keratinocyte-SFM (Thermo Fisher Scientific). The HaCaT cell lines were cultured in a medium with Dulbecco's modified Eagle's medium (DMEM) and fetal bovine serum (FBS) at 37 °C with 5% carbon dioxide. Cells that had reached 40-60% confluence were stimulated with plasma-derived exosomes (100 μg/mL). After 48 h, the cells and supernatants were collected to determine the malondialdehyde (MDA), NAD(P)H, CoQ10 and detect ferroptosis biomarkers in western blotting assays.

### Plasma exosome isolation and characterization

Plasma was mixed with phosphate-buffered saline (PBS) and centrifuged at 1,600 × *g* and 4 °C for 20 min to separate cell debris and macromolecules from exosomes. Supernatants were collected in 26.3 mL ultraclear tubes (Beckman Coulter Inc., Brea, CA, USA) and centrifuged at 10,000 ×* g* and 4 °C for 20 min. The supernatants were passed through a 0.22 μm filter (Millex GP; Darmstadt, Germany) and ultracentrifuged at 120,000 × *g* and 4 °C for 60 min. The exosome pellets were washed in copious ice-cold PBS, centrifuged at 120,000 × *g* and 4 °C for 60 min, resuspended in PBS and stored at -80 °C until use.

Three microliters of exosomes were suspended in PBS, placed on a glow-discharged formvar carbon-coated grid, and negatively stained with 2% (w/v) uranyl acetate. Images were captured with an H-7500 electron microscope (Hitachi, Tokyo, Japan). Particle size was assessed by NanoSight particle tracking (NanoSight, Amesbury, UK). Protein markers of exosomes CD9, CD63, and CD81 were examined by western blotting and quantified by Nano-Flow Cytometry (NanoFCM) utilising a NanoAnalyzer U30 instrument with dual 488/640 nm lasers. Data processing was performed using NanoFCM Professional Suite v1.8 software.

### Expression vector construction and transient transfection

FSP1 and FTH1cDNA was synthesized by Huzhou Hippobiotec Co., Ltd. Small interfering RNAs (siRNAs) against *FSP1*, *NCOA4,* and* FTH1* were purchased from Huzhou Hippobiotec Co., Ltd. The sequences are listed in **Table S3**. A *miR-375-3p* mimic and an inhibitor were purchased from RiboBio Co., Ltd. The following sequence of the *hsa-miR-375-3p* mimic and the inhibitor were used: *hsa-miR-375-3p* mimic, sense (5ʹ-3ʹ), UUUGUUCGUUCGGCUCGCGUGA, and anti-sense (5ʹ-3ʹ), UCACGCGAGCCGAACGAACAAA, and *hsa-miR-375-3p* inhibitor, sense (5ʹ-3ʹ), UCACGCGAGCCGAACGAACAAA. Human primary keratinocytes were plated at 2 × 10^5^ cells/mL into 6-well plates and cultured until reaching approximately 70% confluence. Transient transfection of siRNA or the *miR-375-3p* mimic and inhibitor were carried out using lipofectamine 3000 (Invitrogen, CA, USA). The transfection reagent complexes were added to each well that contained siRNA or the* miR-375-3p* mimic and inhibitor at a final concentration of 10 pM and were then incubated at 37 °C under a 5% CO_2_ atmosphere for 48 h.

### Luciferase reporter assay

The HaCaT cells were seeded into a 24-well plate, and lipofectamine 3000 (Invitrogen, Carlsbad, CA, USA) was used to cotransfect them with luciferase reporter constructs encoding either wild-type *FSP1*, *FTH1, GCH1*, and* DHODH* 3ʹ-UTRs (*FSP1*-, *FTH1*-, *GCH1-*, and *DHODH*-3ʹ UTR-WT) or mutated *FSP1*, *FTH1*, *GCH1*, and *DHODH* 3ʹ-UTR regions (*FSP1*-, *FTH1*-, *GCH1*-, and *DHODH* 3ʹ-UTR-MUT) (GeneChem, Shanghai, China) and either the *miR-375-3p* mimic or a negative control mimic (NC mimic). Luciferase activity was measured with a Dual-Luciferase Reporter Assay kit according to the manufacturer's protocol.

### Loading *miR-375-3p* and siRNA into exosomes

Briefly, 50 μg of exosomes isolated from human plasma of SJS/TEN patients were incubated with Exo-Fect™ siRNA/miRNA transfection kit reagent (System Biosciences, CA, USA), the *miR-375-3p* mimic or inhibitor (10 pM) and *NCOA4, FSP1* or *FTH1* siRNA (10 pM) at 37 °C for 10 min, placed on ice for 30 min, washed 3× with PBS and centrifuged at 2,000 × *g* and 4 °C for 30 min. The transfected cells with exosomes were used in subsequent experiments. Exosome release was blocked with 10 µM GW4869 (Umibio, Shanghai, China).

### Immunohistochemical (IHC) staining

IHC staining was performed overnight at 4 °C as described previously using rabbit polyclonal anti-FSP1 (diluted 1:400, PA5-28727, Thermo), mouse monoclonal anti-4-hydroxynonenal (4-HNE) (diluted 1:100, ab48506, Abcam), rabbit monoclonal anti-transferrin receptor (TFRC; diluted 1:400, ab214039, Abcam), rabbit monoclonal anti-six-transmembrane epithelial antigen of the prostate 3 (STEAP3) (diluted 1:100, D164478, sangon biotech), rabbit monoclonal anti-ACSL4 (diluted 1:200, ab155282, Abcam), rabbit monoclonal anti-ALOX15 (diluted 1:800, ab244205, Abcam), rabbit monoclonal anti-FTH1 (diluted 1:400, ab183781, Abcam), mouse monoclonal anti-8-hydroxy-2′-deoxyguanosine (8-OHdG) (diluted 1:200, ab48508, Abcam), rabbit polyclonal anti-NCOA4 (diluted 1:200, ab111885, Abcam), mouse monoclonal anti-LPCAT3 (diluted 1:200, ab239585, Abcam), mouse monoclonal anti-divalent metal transporter 1 (DMT1) (diluted 1:200, ab55735, Abcam), and rabbit monoclonal anti-IgG isotype control (ab172730, Abcam) antibodies.

### mRNA quantification

For mRNA quantification, cDNA was reverse transcribed with oligo(dT)18 primer, and 18S was used as the internal control. Primers are listed in **Table S4**.

### Antibodies and chemicals

The following antibodies were used in this study: anti-ACSL4 (ab155282; Abcam), anti-glutathione peroxidase 4 (GPX4) (ab125066, Abcam), anti-prostaglandin-endoperoxide synthase 2 (PTGS2) (66351, Proteintech), anti-TFRC (ab214039, Abcam), anti-DMT1 (ab55735, Abcam), anti-NCOA4 (ab86707, Abcam), anti-FTH1 (ab183781, Abcam), anti-FSP1 (PA5-28727, Thermo), anti-ALOX15 (ab244205, Abcam), anti-LPCAT3 (ab239585, Abcam), anti-sterol regulatory element binding protein 1 (SREBP1) (ab28481, Abcam), anti-acyl-CoA 6-desaturase (FADS2) (ab232898, Abcam), anti-fatty acid synthase (FASN) (ab128870, Abcam), anti-stearoyl-CoA desaturase-1 (SCD1) (ab19862, Abcam), anti-Ferroportin1 (FPN1) (ab239583, Abcam), anti-light chain 3B (LC3B) (ab48394, Abcam), anti-4-HNE (ab46545, Abcam), anti-p62 (ab109012, Abcam), anti-ATG5 (ab108327, Abcam), anti-ATG3 (ab108251, Abcam), anti-TAX1BP1 (ab317405, Abcam), anti-Actin, GAPDH, and anti-Tubulin (CW0096M, CW0100M, and CW0098M, CWBIO, Peking, China). Erastin (5 µM) (#S7242), Deferoxamine (DFO) (10 µM) (#S6849) was obtained from Selleck (Houston, TX, USA). Ferrostatin-1 (2 μM) (#T6500) was obtained from TargetMol (Houston, TX, USA). Vitamin E (10 µM) (#V-021), 3-Methyladenine (3-MA) (5 µ M) (HY-19312), and N-acetylcysteine (NAC) (100 µM) (HY-B0215) were obtained from MedChemExpress. The human primary keratinocytes were treated with the indicated reagents for 24 h.

### Immunoblotting

Human primary keratinocytes and HaCaT cells were lysed in denaturing cell extraction buffer. Cell lysates were resolved by sodium dodecyl sulfate-polyacrylamide gel electrophoresis and transferred to a PVDF membrane (Bio-Rad). The membranes were blocked with 5% nonfat milk and probed with the indicated antibodies. HRP-conjugated goat secondary antibodies were used (diluted 1:5,000, Invitrogen). Immunodetection was achieved with Hyglo chemiluminescence reagent (Denville Scientific) and detected with a Bio-Rad ECL machine.

### Cell viability assays and measurements of intracellular iron

Cell viability was determined by Cell Counting Kit-8 (Dojindo, Tokyo, Japan) assay. Labile iron pool (LIP) assay was measured by using calcein acetoxymethyl ester (Corning Inc., Corning, NY, USA) and iron chelator, deferoxamine (Abcam, Cambridge, UK). Intracellular iron contents were also quantified using an iron assay kit (Sigma-Aldrich, St. Louis, MO, USA). FerroOrange (Dojindo) was used to stain intracellular ferrous iron using a 5 μM FerroOrange solution.

### Measurements of free fatty acid, MDA, and oxidized low density lipoprotein (oxLDL)

The relative free fatty acid, MDA and oxLDL levels in the human primary keratinocytes and serum were detected using a free fatty acid (cat. no. ab65341), lipid peroxidation (cat. no. ab118970) and oxLDL (cat. no. ab242302 and cat. no. ab285269) (both from Abcam) assay kits, respectively, according to the manufacturer's instructions.

### Intracellular ROS detection

Dichloro-dihydro-fluorescein diacetate (DCFH-DA, 10 μM) was treated with human primary keratinocytes for 30 min in the dark at 37 °C. After rinsing three times with buffer solution, the intracellular ROS concentration was checked using Fluorescence Microplate Reader (Thermo Fisher Scientific).

### Lipid peroxidation assay

Lipid peroxidation levels were determined with a lipid peroxidation assay kit (ab243377, Abcam) according to the manufacturer's instructions. Briefly, human primary keratinocytes were seeded in dishes and treated with test compounds. Next, a lipid peroxidation sensor was added to the cells and incubated for 30 min at 37 °C. Then, the cells were visualized under an Olympus confocal microscope.

### Quantification of NAD(P)H

The intracellular NAD(P)H level was measured using a NAD(P)H assay kits (Abcam, ab65348 and ab65349) according to the manufacturer's protocol.

### RNAScope® assay

The RNAScope® probe targeting *miR-375-3p* was designed and synthesized by Advanced Cell Diagnostics, and detection of *miR-375-3p* expression was performed using an RNAscope® 2.0 High Definition (HD)-BROWN assay kit according to the manufacturer's instructions (Advanced Cell Diagnostics). The images were acquired with an Olympus confocal microscope.

### Transcriptome sequencing and gene expression analysis

RNA sequencing (RNA-Seq) and gene expression analyses were performed by BioNovoGene (Suzhou, China). Three randomly selected samples in each group were subjected to RNA-Seq experiments. After cluster generation, transcriptome sequencing was carried out on an Illumina Novaseq™ 6000 platform that generated raw reads. The data that support the findings of this study have been deposited in the CNSA (https://db.cngb.org/cnsa/) of CNGBdb with accession number CNP0000970. After removing adaptor sequences, ambiguous'N'nucleotides (with the ratio of 'N' greater than 5%) and low-quality sequences (with a quality score less than 10), the remaining clean reads were assembled using Trinity software as described for de novo transcriptome assembly with a reference genome. The mapped clean-read number was normalized to RPKM (reads per kilo of per million mapped reads). We used the edgeR package to determine the StringTie genes. Threshold of significant difference was |log2foldchange | ≥1, p < 0.05. The Gene Ontology (GO) enrichment analysis, Kyoto Encyclopedia of Genes and Genomes (KEGG) pathway, Heatmap analysis, and VolcanoPlot analysis were conducted at https://www.lc-bio.cn/ (BioNovoGene).

### Analysis of oxylipins

Oxylipins were analyzed using UHPLC-MS/MS with a Shim-pack CBM30A UHPLC system (Shimadzu) connected to a QTRAP MS/MS system (Applied Biosystems) equipped with an electrospray ion source. Lipids were separated using a 1.8 µm C18 column (Agilent). Calibration curves for each oxylipin analyzed were produced using standards from Cayman Chemicals.

### Nontargeted lipidomics analysis by UHPLC-QTOF/MS platform

Phospholipid species were measured by the Shanghai Profleader Biotech Company (Shanghai, China). Lipidome was analyzed by a 1290 UHPLC (Agilent 1290 Infinity II LC Systems) coupled to a TripleTOF 6600 QTOF mass analyzer equipped with a DuoSpray ion source in positive ion mode [electrospray ionization positive (ESI+)] and negative ion mode (ESI-), respectively (SCIEX, Canada). The chromatographic separation was performed using a Kinetex C18 column (2.1 mm by 100 mm, 1.7 μm; Phenomenex) at a flow rate of 300 μl/min and a temperature at 55 °C. Quality control (QC) sample was obtained by pooling all prepared samples and served to equilibrate chromatographic system and correct variations during lipidomics instrumental analysis. The mobile phases comprised (A) acetonitrile:water (60:40, v/v) with 10 mM ammonium formate and (B) isopropanol:acetonitrile (90:10, v/v) with 10 mM ammonium formate. A linear gradient elution was performed with the following procedure: 0 min, 40% B; 12 min, 100% B; 13.5 min, 100% B; 13.7 min, 40% B and held to 18 min. Eight runs of QC sample was conducted to balance chromatographic system before formal determination, and one run of QC sample was inserted when three samples were analyzed.

The main parameters of MS were as follows. Ion source temperature was 600 °C, and ion spray voltage floating (ISVF) was set to 5000 V (ESI+) or 4500 V (ESI-). The pressures of nebulizer gas, heater gas, and curtain gas were set to 60, 60, and 30 psi, respectively. A typical information-dependent acquisition including TOF MS scan and then tandem MS (MS/MS) experiment was performed in the analysis. The TOF MS scan was performed under high-resolution settings with a range of 200 to 2000 mass/charge ratio (m/z) and an accumulation time of 200 ms. The declustering potential and collision energy (CE) were set at 100 V and 10 eV for ESI+ or at -100 V and -10 eV for ESI-, respectively. In the second experiment, top 10 candidate precursors per scan cycle were fragmented in collision-induced dissociation by a CE setting at 45 eV (ESI+) or -45 eV (ESI-) both with CEs of 25 eV, and the data were collected at a range of 100 to 2000 m/z with 50-ms accumulation time for the products of each precursor. The software for controlling instrument and acquiring data was Analyst TF 1.7.1 (SCIEX, Comcord, Ontario, Canada).

### Statistical analysis

Data were subjected to Mann-Whitney *U* test and one-way ANOVA to compare group means. The data were tested for normality prior to performing parametric assessments, which were conducted with GraphPad Prism software version 6 (GraphPad software). Pearson's correlation coefficients were evaluated. *P* < 0.05 was considered to be statistically significant. The significance level (*P* value) for Pearson's correlation coefficient (*R*) was obtained by *t* test, where 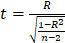 with degrees of freedom (df) = n - 2. All data are reported as the means ± SEM on the basis of triplicate values obtained from three experiments.

## Results

### The skin lesions and plasma of SJS/TEN patients exhibit elevated levels of lipid peroxidation and iron

**Figure 1:**
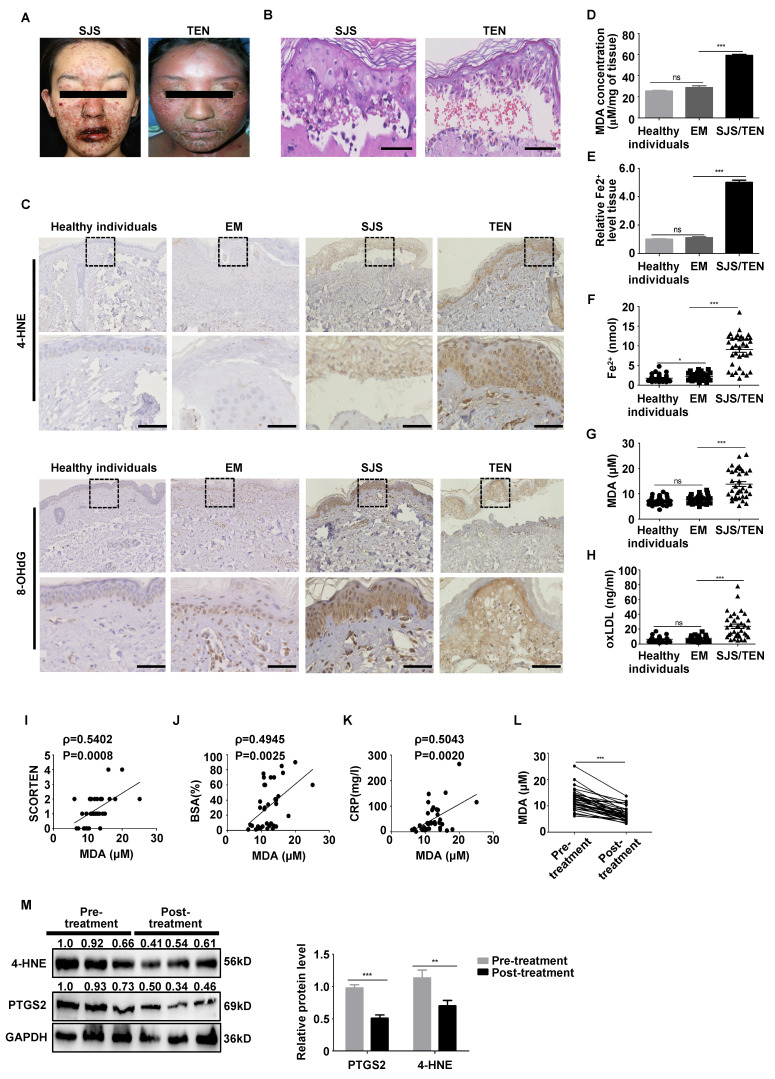
** The skin lesions and plasma of patients with SJS/TEN had increased lipid peroxidation and iron levels.** (**A**) Image of a patient with SJS/TEN with facial erythematous macules, accompanied by erosions and crusts of the eyelid and lips. (**B**) Hematoxylin and eosin (H&E) staining shows skin lesions of patients with SJS/TEN. (**C**) 4-HNE and 8-OHdG immunostaining. (**D-E**) MDA and iron levels in skin lesions of patients with SJS/TEN. (**F**) The intracellular iron level. (**G**) MDA level. (**H**) oxLDL levels in the plasma of patients with SJS/TEN. (**I-K**) MDA level correlated positively with the disease severity of SCORTEN (**I**), BSA (**J**) and CRP (**K**) in patients with SJS/TEN. (**L-M**) The levels of MDA (**L**), 4-HNE, and PTGS2 (**M**) in patients with SJS/TEN before and after treatment with system corticosteroids at the onset of disease. *Bar* = 100 µm. *ns*, not significant, *P < 0.05, **P < 0.01, ***P < 0.001.

Patients with SJS/TEN exhibit multiple erythematous macules evolving into bullae and epidermal detachment on the face/lips (**Fig. 1A**). The histopathology of SJS/TEN is characterized by a subepidermal cleft with overlying confluent necrosis of the epidermis (**Fig. 1B**). First, we measured several putative biomarkers of lipid peroxidation, including the 8-OHdG oxidative product 23 and 4-HNE modifications, which are the two main byproducts of lipid peroxidation 24. IHC revealed significant 4-HNE and 8-OHdG upregulated expression in SJS/TEN lesions relative to those of patients with EM or MPE and healthy individuals (**Fig. 1C and Fig. S1A-B**). We also observed upregulated expressions of the iron importers TFRC, LPCAT3, and DMT1 25 in the skin lesions of patients with SJS/TEN (**Fig. S2**). In addition, the skin lesions and plasma of patients with SJS/TEN showed dramatically higher iron levels, MDA and oxLDL than EM and healthy individuals (*P* < 0.05) (**Fig. 1D-H**). Then, The MDA accumulation correlated positively with the disease severity of the severity-of-illness score for toxic epidermal necrolysis (SCORTEN), body surface area (BSA) and C-reactive protein (CRP) in patients with SJS/TEN (*P* < 0.05) (**Fig. 1I-K**). The levels of MDA, 4-HNE and PTGS2 reduced significantly in the plasma and skin lesions of patients with SJS/TEN after 2 weeks of system corticosteroids treatment (*P* < 0.05) (**Fig. 1L-M**). These data suggest that ferroptosis may be involved in the pathogenesis of SJS/TEN.

### Plasma-derived exosomes characterization

Plasma exosomes were isolated and observed using ultra-centrifugation and transmission electron microscopy (TEM), respectively (**Fig. S3A**). Immunoblotting was performed to characterize cells stained for exosomal markers (**Fig. S3B**). Nano-Flow Cytometry (NanoFCM) indicated that the exosomes were positive for CD9, CD63 and CD81 markers (**Fig. S3C**). Nanoparticle tracking analysis determined particle size distribution in purified, exosome-rich preparations. The average particle sizes in normal, EM, and SJS/TEN plasma lied within the expected 30-200 nm range (**Fig. S3D**).

### Plasma-derived exosome from SJS/TEN patients induce keratinocyte ferroptosis

**Figure 2:**
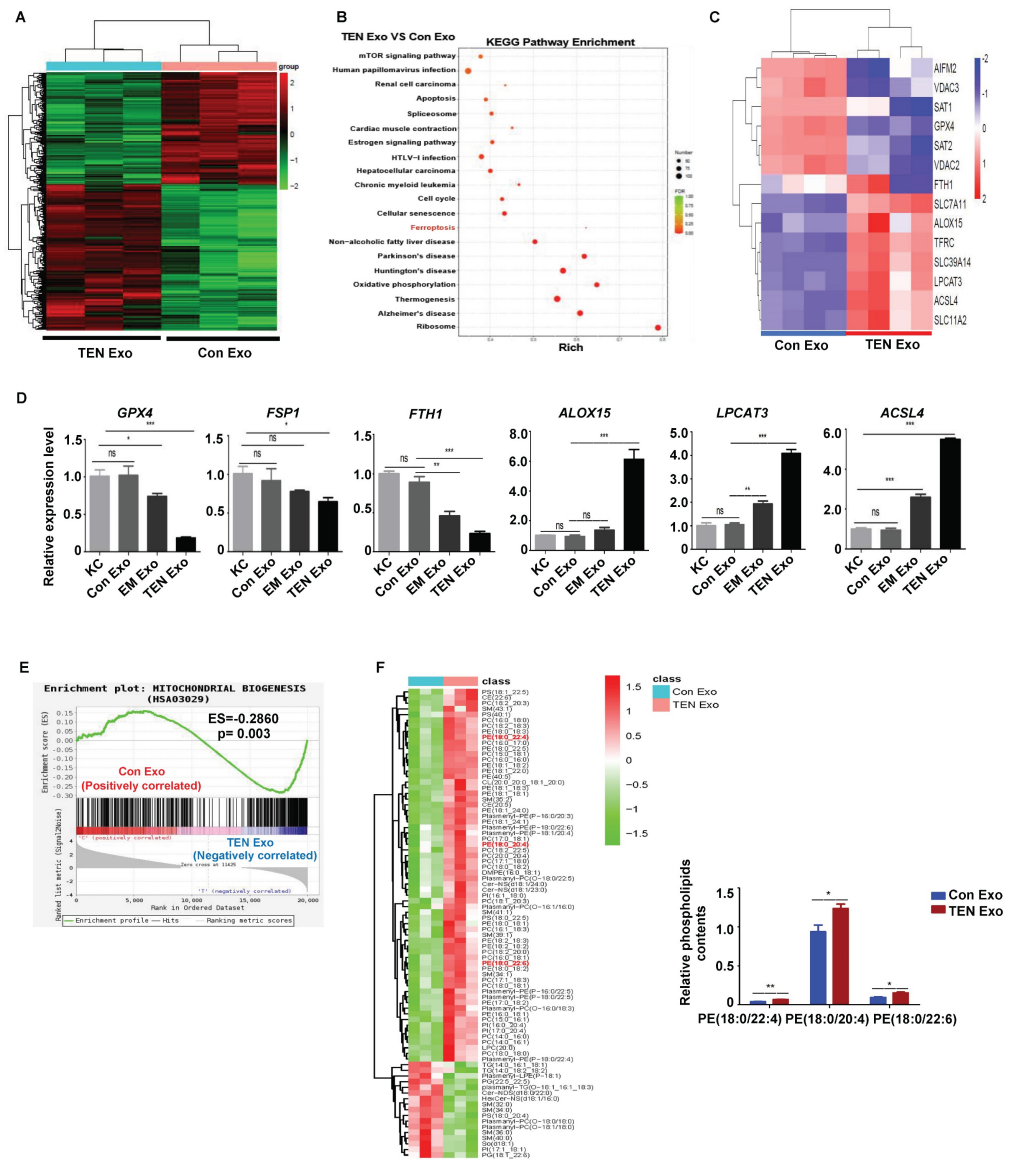
** Transcriptome and lipidomics analysis.** (**A**) DEGs between Con Exo and TEN Exo; n=3 patients, and N=3 technical replicates per group. We repeated the experiments for at least 3 times, with at least 3 individuals or specimens in each group. (**B**) Kyoto Encyclopedia of Genes and Genomes (KEGG) pathway between TEN Exo and Con Exo groups. (**C**) Heatmap of ferroptosis DEGs between Con Exo and TEN Exo groups. (**D**) *GPX4*, *FSP1*, *ACSL4*, *ALOX15*, *FTH1* and *LPCAT3* mRNA levels were analyzed using qRT-PCR. (**E**) Gene set enrichment analysis (GSEA) of DEGs between TEN Exo and Con Exo groups. (**F**) Heatmap of phospholipid changes between TEN Exo and Con Exo groups as assessed via non-targeted metabolomics analysis. *ns*, not significant, *P < 0.05, **P < 0.01, ***P < 0.001.

The RNA-Seq analysis was performed to identify differentially expressed genes (DEGs) in the keratinocyte of the exosome-treated group to investigate better the effect of plasma-derived exosome from patients with SJS/TEN. The results revealed 345 upregulated and 266 downregulated DEGs between the TEN Exo and Con Exo groups, based on a fold change >2 and *P* < 0.05 (**Fig. 2A**). Furthermore, the ferroptosis pathway was one of the pathways most significantly enriched with DEG in the TEN Exo group, suggesting that ferroptosis may be involved in keratinocyte death (**Fig. 2B**). Subsequently, hierarchical clustered heatmaps of DEGs in the ferroptosis pathway were generated (**Fig. 2C**). In addition, we validated the expression of ferroptosis genes through RNA-Seq and observed consistent trends (**Fig. 2D**). Ferroptosis is indeed associated with severe damage to mitochondrial biogenesis 5. A gene set enrichment analysis (GSEA) revealed that mitochondrial biogenesis was disrupted in the TEN Exo group (**Fig. 2E**). Accordingly, the levels of phospholipids PE (18:0/20:4), (18:0/22:4), and (18:0/22:6), which are key phospholipids that undergo oxidation and drive cells toward ferroptotic death 8, increased in the TEN Exo group (*P* < 0.05) (**Fig. 2F**). All these data provide comprehensive evidence that plasma-derived exosome from patients with SJS/TEN may induce keratinocyte ferroptosis.

**Figure 3:**
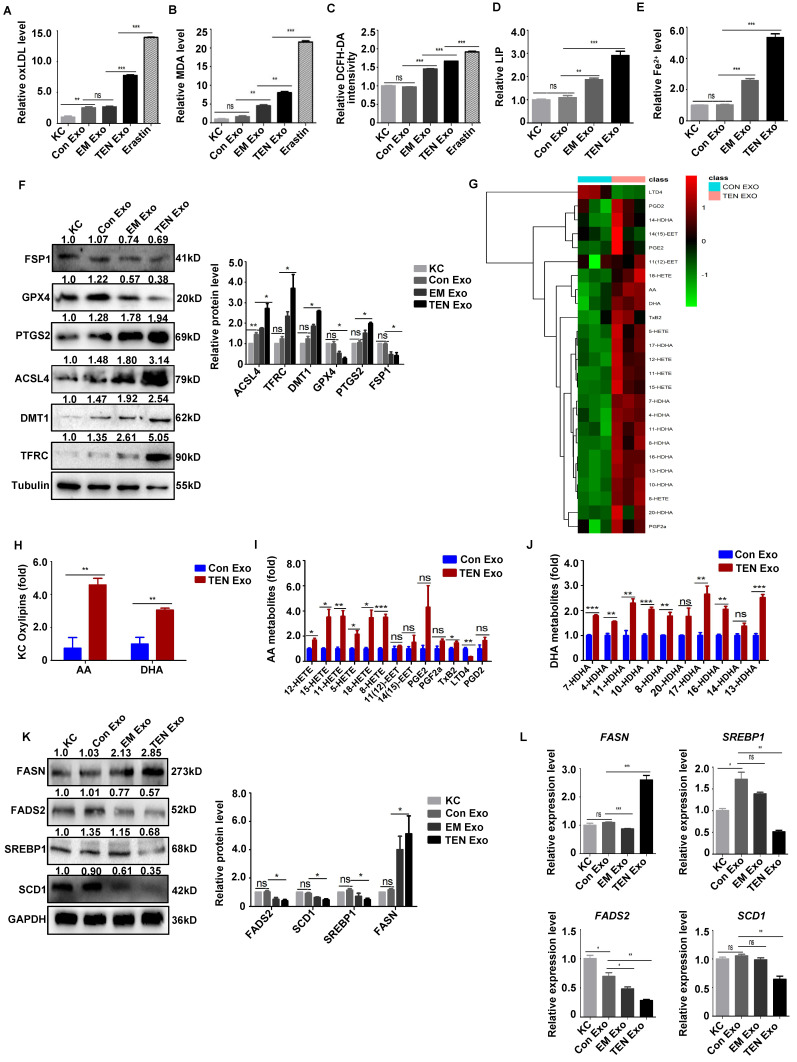
** Plasma-derived exosome induced keratinocyte ferroptosis.** (**A-L**) Human primary keratinocytes were incubated with Con Exo, EM Exo, TEN Exo (100 μg/mL), or erastin (5 μM). (**A**) oxLDL assay. (**B**) MDA assay. (**C**) Intracellular ROS levels. (**D**) LIP levels. (**E**) Fe^2+^ accumulation. (**F**) FSP1, ACSL4, PTGS2, DMT1, TFRC, STEAP3, and GPX4 protein expressions were analyzed by western blotting. (**G**) Levels of oxylipin. (**H-J**) Summary of keratinocyte oxylipins, arachidonic acid (AA) metabolites, and docosahexaenoic acid (DHA) metabolites. (**K-L**) The protein and mRNA levels of FASN, FADS2, SCD1, and SREBP1 in keratinocyte. The data are reported as the means ± SEMs of three independent experiments. *ns*, not significant. *P < 0.05, **P < 0.01, ***P < 0.001.

First, we revealed that most of the exosomes had been internalized by keratinocyte (**Fig. S4A**) to investigate further whether keratinocyte internalize plasma-derived exosome to promote ferroptosis in SJS/TEN. Subsequently, we measured oxLDL, MDA, intracellular ROS, labile iron pool (LIP), and iron accumulation, indicators of key signaling events in ferroptosis induction. The oxLDL, MDA, intracellular ROS, LIP, and intracellular Fe^2+^ levels increased in keratinocyte following TEN Exo treatment (*P* < 0.05) (**Fig. 3A-E**). Furthermore, TEN Exo induced the expression of ferroptosis biomarkers in keratinocyte (*P* < 0.05) (**Fig. 3F**). Interestingly, the exosomes did not harbor ferroptosis biomarkers (**Fig. S4B**). Lipid peroxidation levels were notably elevated in the TEN Exo treatment relative to the Con Exo treatment. (*P* < 0.05) (**Fig. S4C**). The induction of ferroptosis necessitates the oxidation of PUFA, forming precursors for bioactive oxylipins 8. We discovered that the levels of keratinocyte oxylipins arachidonic acid (AA) and docosahexaenoic acid (DHA) were significantly upregulated in the TEN Exo group (**Fig. 3G-H**), and oxidized AA metabolites 12-hydroxyeicosatetraenoic (HETE), 11-HETE, 8-HETE, 15-HETE, TxB2, LTD4 and oxidized DHA metabolites 7-HDHA, 4-HDHA, 11-HDHA, 10-HDHA in TEN Exo treatment increased (*P* < 0.05) (**Fig. 3I-J**). FADS2, SCD1, and SREBP1 are key determinants of cellular sensitivity to ferroptosis 7. Then, we concluded that FADS2, SCD1, and SREBP1 decreased and FASN increased in the cells with TEN Exo treatment (**Fig. 3K-L**). Besides, IHC results revealed that ALOX15, ACSL4, and LPCAT3 were significantly upregulated in SJS/TEN skin lesions relative to those of patients with EM or MPE and healthy individuals (**Fig. S5**). Together, these findings indicate that TEN Exo strongly promotes sensitivity to ferroptosis via intracellular ferrous iron accumulation, PUFA generation, and lipid peroxidation.

### ROS scavengers and lipophilic antioxidants significantly inhibit keratinocyte ferroptosis after TEN Exo treatment

**Figure 4:**
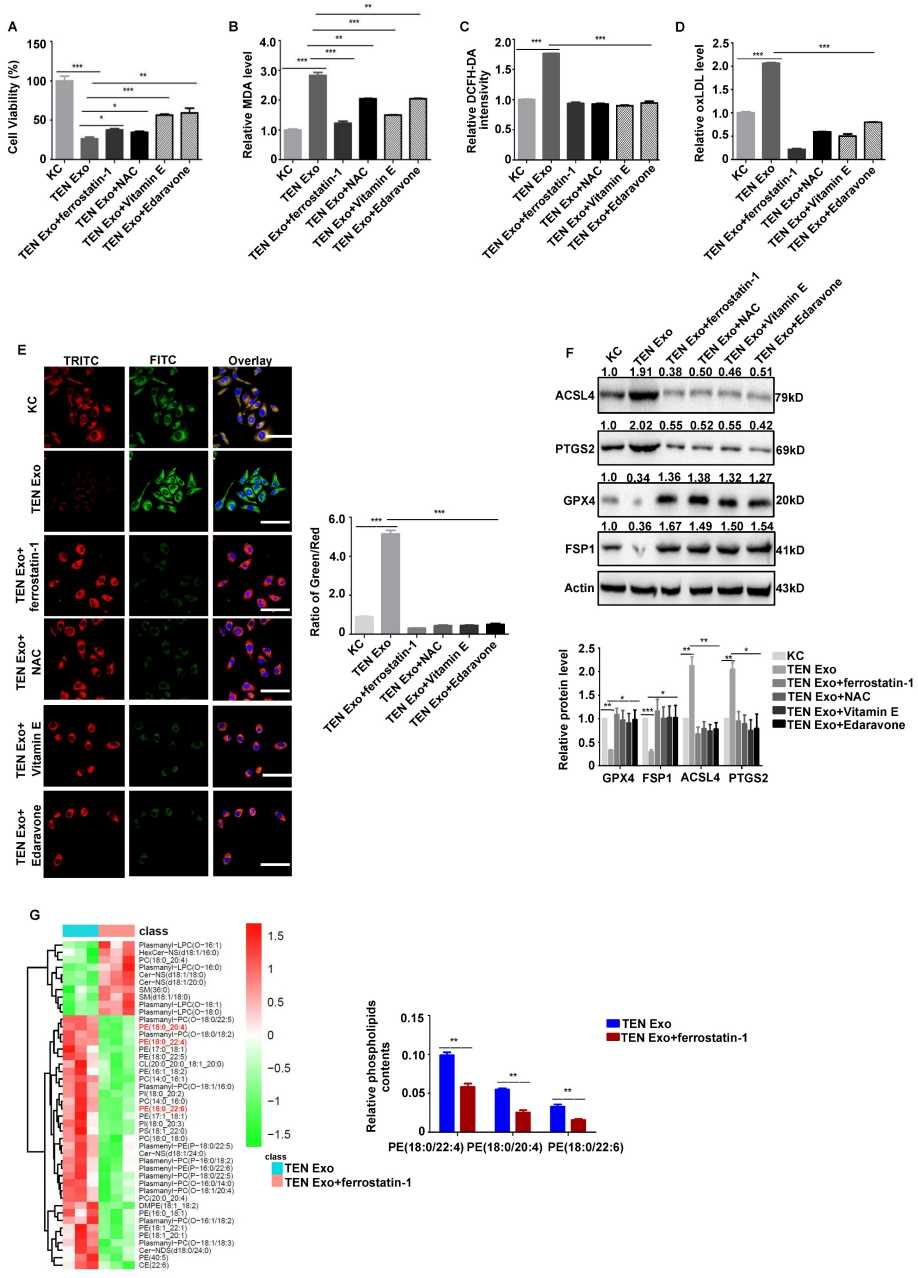
** ROS scavengers and antioxidants significantly inhibit keratinocyte ferroptosis treated with TEN Exo.** (**A-F**) Human primary keratinocytes were treated with TEN Exo, ferrostatin-1 (2 µM), and ROS scavengers (10 mM NAC; 5 µM vitamin E; 200 µM edaravone). (**A**) Cell viability was analyzed by CCK-8 assay. (**B**) Lipid peroxidation. (**C**) Intracellular ROS levels. (**D**) oxLDL accumulation. (**E**) Lipid peroxidation accumulation. *Bar* = 100 µm. (**F**) ACSL4, PTGS2, FSP1, and GPX4 protein expression were analyzed by western blotting. (**G**) Heatmap of phospholipid changes between TEN Exo and TEN Exo plus ferrostatin-1 group as assessed via nontargeted metabolomics analysis. The data are reported as the means ± SEMs of three independent experiments. *ns*, not significant. *P < 0.05, **P < 0.01, ***P < 0.001.

Human primary keratinocytes were cotreated with ROS scavengers and lipophilic antioxidants to determine whether exosome-induced ROS accumulation was the key factor contributing to ferroptosis in SJS/TEN. The results revealed that ROS scavengers (NAC, vitamin E, and edaravone) and ferroptosis inhibitor ferrostatin-1 significantly inhibited TEN Exo-induced increase in cell viability, MDA, intracellular ROS, oxLDL, lipid peroxidation, and ferroptosis biomarkers (*P* < 0.05) (**Fig. 4A-F**). Ferrostatin-1 also substantially decreased the levels of keratinocyte phospholipids PE (18:0/20:4), (18:0/22:4), and (18:0/22:6) (*P* < 0.01) (**Fig. 4G**). Hence, ROS scavengers and antioxidants significantly inhibit keratinocyte ferroptosis after TEN Exo treatment.

### NCOA4 promoted the degradation of FTH in TEN Exo-induced keratinocyte

Ferritinophagy was closely related to the increased levels of lipid peroxidation 12. Therefore, we tested whether ferritinophagy is involved in SJS/TEN pathogenesis. First, we detected the level of ferritinophagy biomarkers in keratinocyte after TEN Exo treatment. The data demonstrated that TEN Exo upregulated NCOA4 expression but downregulated FTH1 expression (*P* < 0.05) (**Fig. S6A**). Subsequently, after TEN Exo stimulation for the indicated times, FTH1 levels decreased gradually, while NCOA4 and intracellular iron levels increased gradually (**Fig. S6B-C**) and the iron chelator DFO reduced intracellular iron levels (**Fig. S6D**). In addition, we detected whether the other autophagy markers involved in the pathogenesis of SJS/TEN patients. The results showed that NCOA4 and FTH1 expression levels were the most upregulated and downregulated in keratinocyte after TEN Exo treatment relative to the other autophagy markers respectively. Meanwhile, after treatment with the autophagy inhibitors 3-MA, only the NCOA4 and FTH1 expression levels have been significantly restored (*P < 0.01*) (**Fig. S6E**). Therefore, these results demonstrated that the ferritinophagy is specifically involved in SJS/TEN pathogenesis. Subsequently, the results revealed that NCOA4 knockdown mitigated the TEN Exo-induced keratinocyte ferroptosis, as evidenced by decreased PTGS2 and ACSL4 and increased FTH1 levels (**A-D**), oxLDL (**E**), intracellular ROS (**F**), MDA (**G**), and lipid ROS (**H**) (*P* < 0.05) (**Fig. S7A-H**). *In vivo*, IHC revealed significant NCOA4 upregulation and FTH1 downregulation in SJS/TEN skin lesions relative to those in patients with EM or MPE and healthy individuals (**Fig. S8**). Hence, these data indicate that NCOA4-mediated ferritinophagy is required for the keratinocyte ferroptosis induced by TEN Exo stimulation.

### *miR-375-3p* promotes keratinocyte ferroptosis and contributes to SJS/TEN pathogenesis

We previously used miRNA sequencing to compare the miRNA profiles between the TEN Exo and Con Exo groups. Notably, we discovered that* miR-375-3p* was markedly upregulated in TEN Exo relative to the other groups 19. Furthermore, RNAscope *in situ* hybridization revealed that *miR-375-3p* signals were only expressed in the epidermal tissues of patients with SJS/TEN (**Fig. S9**). Therefore, we focused on *miR-375-3p* in our research. Subsequently, we tested whether exosomal *miR-375-3p* was involved in keratinocyte ferroptosis in patients with SJS/TEN. Initially, we revealed that keratinocyte internalized TEN Exo loaded with *miR-375-3p* (**Fig. S10A**). Treatment with the exosome-specific inhibitor GW4869 significantly downregulated the keratinocyte *miR-375-3p* levels (*P* < 0.05) (**Fig. S10B**). Therefore,* miR-375-3p* transfer from the plasma to keratinocyte is exosome-dependent. Furthermore, *miR-375-3p*-loaded TEN Exo promoted keratinocyte ferroptosis, while the *miR-375-3p* inhibitor suppressed keratinocyte ferroptosis (*P* < 0.05) (**Fig. S10C-H**).

**Figure 5:**
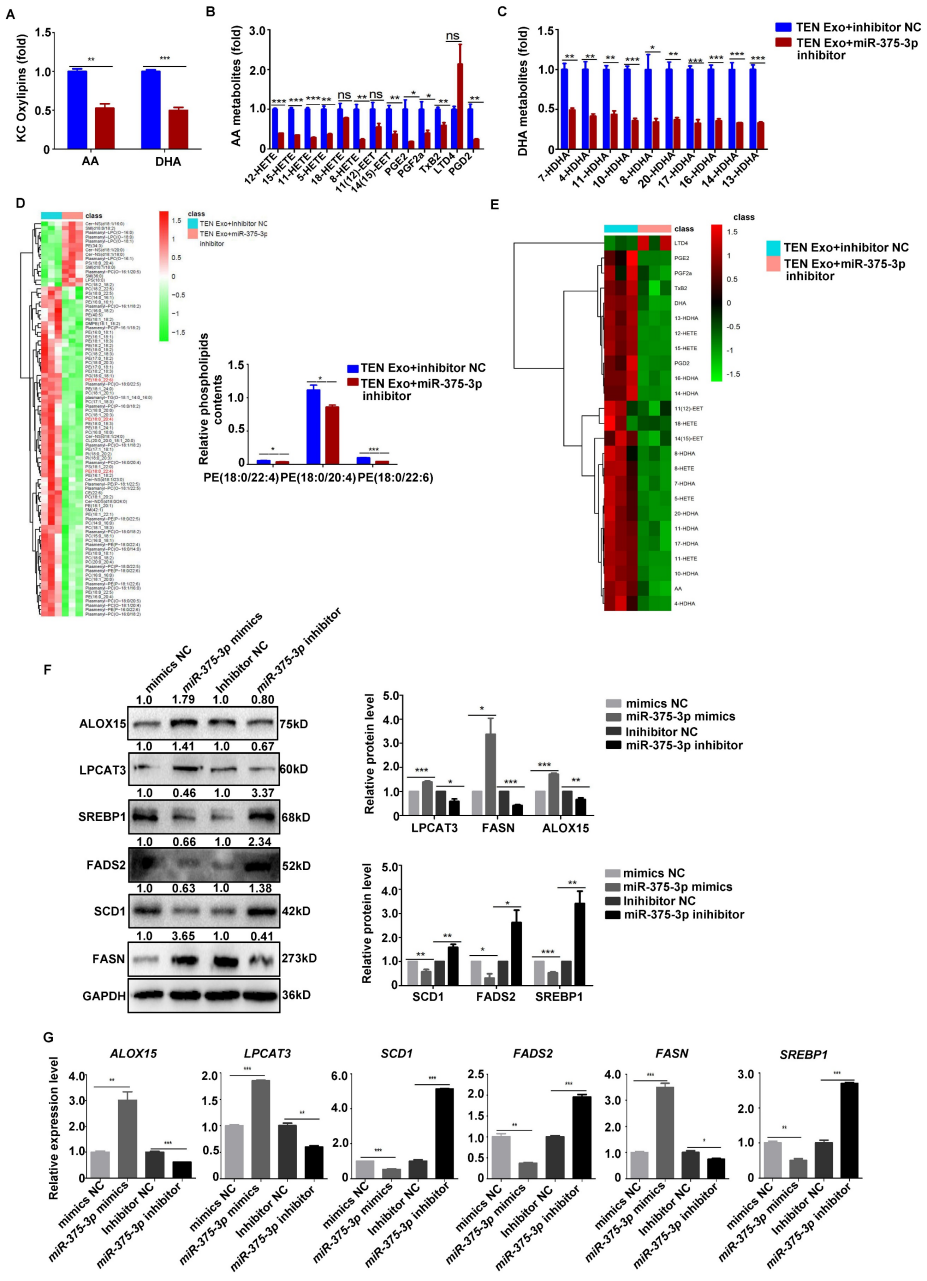
***miR-375-3p* positively regulates keratinocyte lipid peroxidation.** (**A-D**) Summary of keratinocyte oxylipins, AA metabolites, DHA metabolites, and heatmap of phospholipid changes after transfection with an NC inhibitor and a *miR-375-3p* inhibitor. (**E-G**) Levels of oxylipin, ALOX15, LPCAT3, SCD1, FASN, FADS2, and SREBP1 mRNA and protein expressions after transfection with *miR-375-3p* mimics or inhibitor. The data are reported as the means ± SEMs of three independent experiments. *ns*, not significant, *P < 0.05, **P < 0.01, ***P < 0.001.

Subsequently, we further measured lipid peroxidation to elucidate the molecular mechanism of *miR-375-3p* action in ferroptosis. The efficiency of the *miR-375-3p* was confirmed using qRT-PCR (**Fig. S11A**). Additionally, our findings indicate that overexpression of *miR-375-3p* resulted in heightened MDA accumulation (**Fig. S11B**), elevated intracellular Fe^2+^ levels (**Fig. S11C, G**), increased levels of LIP, intracellular ROS, lipid peroxidation, oxLDL levels, and ferroptosis modulators (**Fig. S11D, E, F, K, L**), while significantly reducing NAD(P)H (**Fig. S11H-I**) and CoQ10 levels (*P* < 0.05) (**Fig. S11J**). Importantly, *miR-375-3p* inhibition significantly decreased the levels of phospholipids PE (18:0/20:4), (18:0/22:4), and (18:0/22:6), cellular oxidized PUFA (AA and DHA), AA and DHA metabolites (*P* < 0.05) (**Fig. 5A-E**). Overexpression of *miR-375-3p* also upregulated ALOX15, LPCAT3, and FASN, but downregulated FADS2, SCD1, and SREBP1 expression in keratinocyte (*P* < 0.05) (**Fig. 5F, G**). Therefore, *miR-375-3p* promotes keratinocyte ferroptosis and might contribute to SJS/TEN pathogenesis.

### Downregulation of FSP1 and FTH1 by *miR-375-3p* expression contributes to keratinocyte ferroptosis

**Figure 6:**
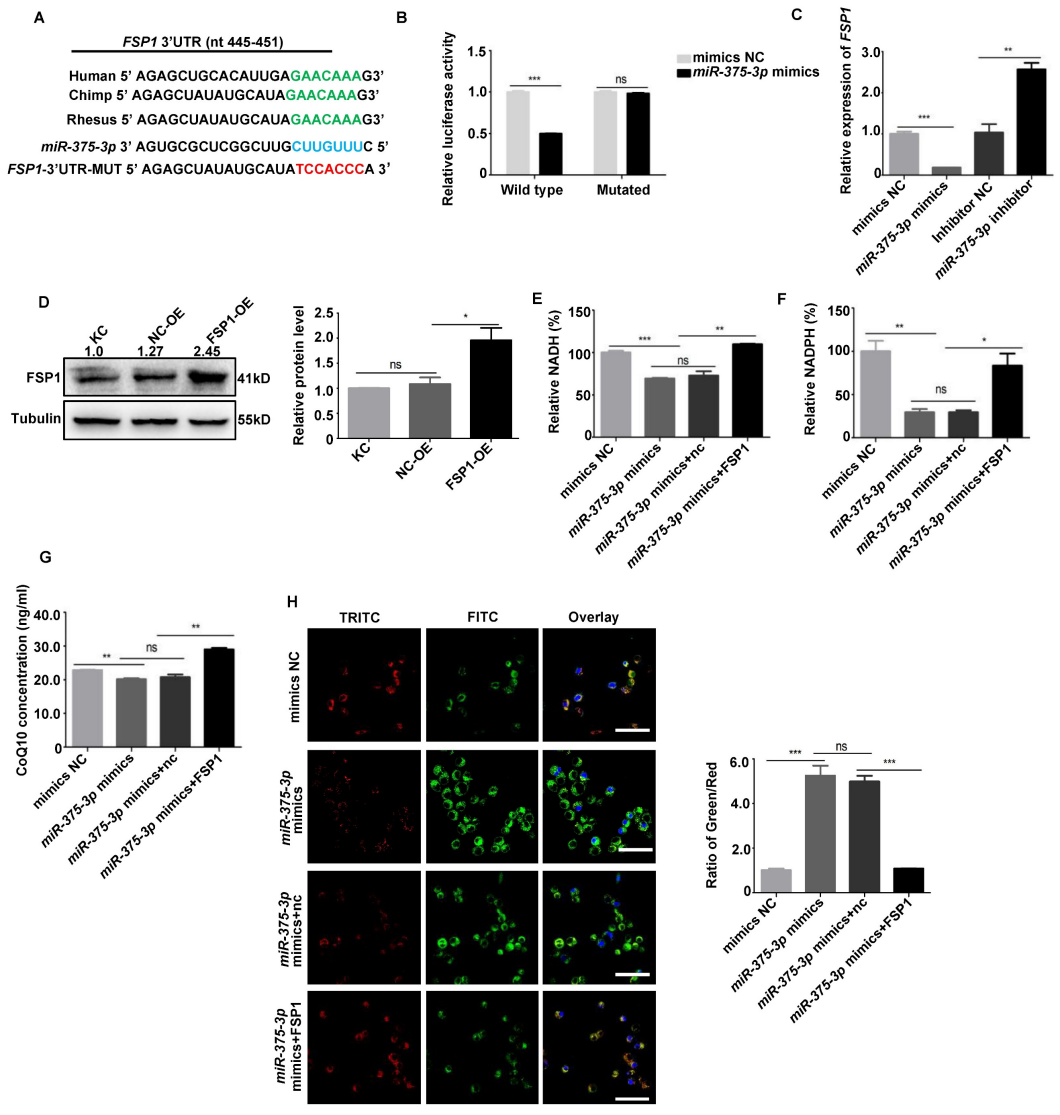
***miR-375-3p* promoted ferroptosis in HaCaT cells by targeting *FSP1*.** (**A**) Schematic diagram showing putative *miR-375-3p* binding sites in the 3ʹ-UTR of human *FSP1*. (**B**) HaCaT cells transfected with wild-type or mutant *FSP1* 3ʹ-UTR luciferase and negative control mimic or *miR-375-3p* mimic constructs. (**C-D**) FSP1 mRNA and protein expression. (**E-H**) Reintroducing FSP1 into HaCaT cells partially rescued ferroptosis mediated by *miR-375-3p*, as determined by NAD(P)H (**E-F**), CoQ10 levels (**G**), and lipid peroxidation accumulation (**H**). The data are reported as the means ± SEMs of three independent experiments. *Bar* = 100 µm. *ns*, not significant. *P < 0.05, **P < 0.01, ***P < 0.001.

**Figure 7:**
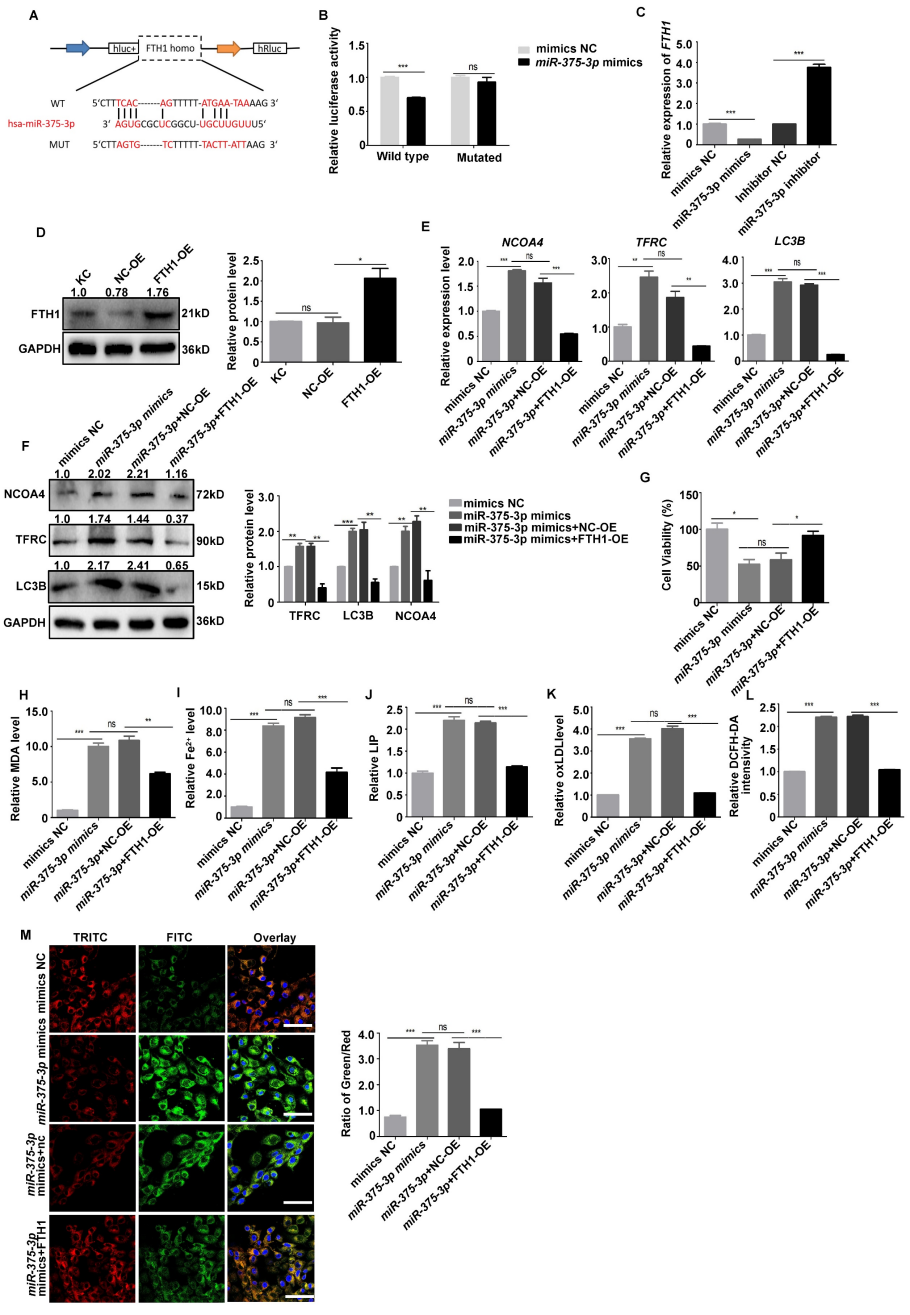
***miR-375-3p* promoted ferroptosis in HaCaT cells by targeting *FTH1*.** (**A**) Schematic diagram showing putative *miR-375-3p* binding sites in the 3ʹ-UTR of human *FTH1*. (**B**) HaCaT cells transfected with wild-type or mutant *FTH1* 3ʹ-UTR luciferase and negative control mimic or *miR-375-3p* mimic constructs. (**C-D**) FTH1 mRNA and protein expression. (**E-F**) NCOA4, TFRC, and LC3B mRNA and protein expression. (**G-M**) Reintroducing FTH1 into HaCaT cells partially rescued ferroptosis mediated by *miR-375-3p*, as determined using the CCK-8 assay (**G**), MDA level (**H**), Fe^2+^ accumulation (**I**), LIP levels (**J**), oxLDL levels (**K**), Lipid peroxidation accumulation (**L**), and intracellular ROS levels (**M**). *Bar* = 100 µm. *ns*, not significant. *P < 0.05, **P < 0.01, ***P < 0.001.

Ferroptosis defense systems mainly include the GPX4-GSH, FSP1-CoQH_2_, dihydroorotate dehydrogenase (DHODH)-CoQH_2_, FTH1, and GTP cyclohydrolase 1 (GCH1)-tetrahydrobiopterin (BH_4_) systems 5. Therefore, we detected whether the above target molecules could bind with *miR-375-3p,* and the data demonstrated that *FSP1* and* FTH1* were potential targets of *miR-375-3p* (**Fig. S12A-C**). The *miR-375-3p* binding sites in the *FSP1* and* FTH1* 3ʹ-UTR were listed in **Fig. 6A and Fig. 7A**. The *miR-375-3p* suppressed luciferase activity in the wild-type but not in the mutant construct (**Fig. 6B and Fig. 7B**). FSP1 and FTH1 expression were downregulated in keratinocyte overexpressing *miR-375-3p* (**Fig. 6C and Fig. 7C**). These results reveal that *miR-375-3p* regulates FSP1 and FTH1 expression by binding to its 3ʹ UTR.

Then, we restored FSP1 and FTH1 expression by transfecting cells harboring the *miR-375-3p* mimic with the FSP1 and FTH1 plasmids to confirm whether FSP1 and FTH1 target *miR-375-3p* to promote keratinocyte ferroptosis (**Fig. 6D and Fig. 7D**). *miR-375-3p*-mediated keratinocyte ferroptosis was partially counteracted in the FSP1 and FTH1-overexpressing cells (**Fig. 6E-H and Fig. 7E-M**). In addition, *FSP1* and *FTH1* knockdown significantly increased keratinocyte ferroptosis (*P* < 0.05) (**Fig. S13A-I and** 14A-K). *In vivo*, IHC revealed significant FSP1 downregulation in SJS/TEN skin lesions relative to those in patients with EM or MPE and healthy individuals (**Fig. S8**). These results revealed that *miR-375-3p* induced keratinocyte ferroptosis by downregulating both FSP1 and FTH1 expression in patients with SJS/TEN. In addition, we tested the potential roles of TNF-α or XIAP in mediating the ferroptosis in human primary keratinocytes. The results demonstrated that there were no observed changes in intracellular ROS, MDA, iron accumulation, lipid peroxidation, or the expression of ferroptosis biomarkers in human primary keratinocytes following TNF-α treatment or *XIAP*-siRNA transfection (**Fig. S15A-E and Fig. S16A-E**).

### Exosomal *miR-375-3p* levels in SJS/TEN patients are positively correlated with biomarkers of ferroptosis

We also demonstrated that circulating exosomal *miR-375-3p* was positively correlated with intracellular Fe^2+^ and MDA levels but negatively correlated with CoQ10 level in SJS/TEN patients (**Fig. S17**). Therefore, circulating exosomal *miR-375-3p* may be a diagnostic biomarker for SJS/TEN.

## Discussion

**Figure 8:**
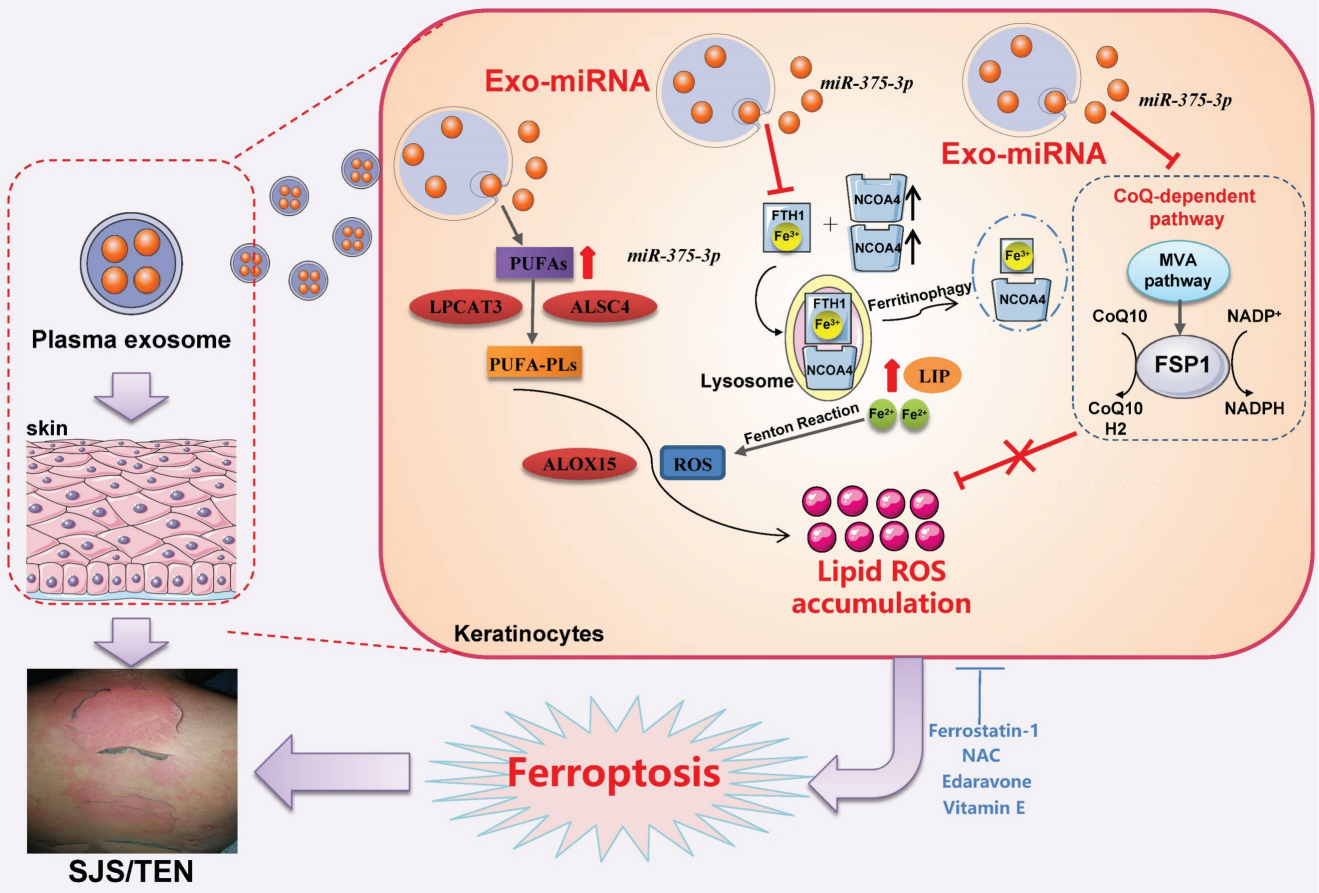
Schematic diagram of *miR-375-3p* mediated lipid metabolism, ferritinophagy and CoQ-dependent pathway contributes to the ferroptosis of keratinocyte in SJS/TEN patients.

Our study demonstrated that the skin lesions and plasma of patients with SJS/TEN had increased lipid peroxidation and iron levels. *miR-375-3p*-loaded TEN Exo upregulated cellular oxidized PUFA and decreased FTH1 expression. The degradation of FTH1 releases iron stored in ferritin into the LIP; consequently, the blockade of NCOA4-mediated ferritinophagy notably suppresses ferroptosis. Therefore, we hypothesized that circulating exosomal *miR-375-3p* entered keratinocyte, promoting cellular oxidized PUFA and substantially downregulating both FSP1 and FTH1 expression, inducing the CoQ-dependent and ferritinophagy-mediated ferroptosis pathway in SJS/TEN (**Fig. 8**). Considering these data, we concluded that ferroptosis inhibitors may be potential therapeutic agents for SJS/TEN treatment in the future.

In previous studies, the pan-caspase inhibitor zVAD and inhibitor of receptor-interacting kinase 1 (RIPK1) necrostatin-1 did not fully rescue keratinocyte death in SJS/TEN, suggesting the potential involvement of alternative forms of cell death in the pathogenesis of SJS/TEN 3-4. Notably, ferroptosis was implicated in the pathological cell death of heart tissues subjected to ischemia/reperfusion injury 6. Moreover, characteristics of ferroptosis, including the loss of glutathione (GSH) and induced lipid peroxidation, have also been reported in Alzheimer's and Parkinson's disease models 26-27. Mounting evidence have reported that sulfasalazine and sorafenib, which increase the risk of SJS/TEN, induced ferroptosis 28. Reduced synthesis of GSH could pose a risk for TEN, with NAC serving as an effective treatment for SJS/TEN 29-30. However, our understanding of the pathogenesis of SJS/TEN has been hindered by the lack of direct evidence for ferroptosis. In this study, we indicated that ferroptosis biomarkers were upregulated in skin lesions of patients with SJS/TEN. The MDA levels in the plasma of patients with SJS/TEN correlated positively with the disease severity of SCORTEN. These data led us to hypothesize that ferroptosis may be associated with SJS/TEN pathological mechanisms.

In various diseases, enhanced exosome production in patients is closely associated with the disruption of cell death 31-34. In this study, we displayed that keratinocyte internalized TEN Exo significantly promoted ferroptosis. MiRNAs are found in secreted exosomes and play important roles in diverse progress of cell death 14-18. Our previous study revealed that *miR-375-3p* promoted keratinocyte apoptosis by repressing *XIAP* in patients with SJS/TEN 19. The functions of *miR-375-3p* in keratinocyte were the foci of this study because of its high potential in SJS/TEN pathogenesis. In this study, exosomal *miR-375-3p* substantially enhanced keratinocyte ferroptosis and was positively correlated with intracellular Fe^2+^ and MDA levels in patients with SJS/TEN. Interestingly, acute pancreatic injury is a gastrointestinal complication of SJS/TEN that increases the risk of hepatotoxicity 35. Moreover, *miR-375-3p* is highly expressed in pancreatic islets and contributes to the injury caused by pancreatic cancer 36. Therefore, pancreatic islets are possible sources of SJS/TEN plasma exosomes. Keratinocytes absorb all circulating exosomal contents, indicating further exploration is necessary before conclusively identifying *miR-375-3p* as the sole key factor. Future research on SJS/TEN can focus on other diagnostic markers to expand our understanding of their pathogeneses and advance translational medicine by designing exosome-based therapeutic applications.

Lipid metabolism is closely related to cell sensitivity in ferroptosis. During ferroptosis, PUFAs, especially AAs, are highly susceptible to peroxidation, thus disrupting membrane function 8. DHA also represents a promising therapeutic agent to induce ferroptosis 37. Therefore, the abundance of PUFAs correlates with the degree of lipid peroxidation, determining the sensitivity of cells to ferroptosis. In this study, TEN Exo treatment and *miR-375-3p* increased cellular oxidized PUFA (AA and DHA), phospholipids PE (18:0/20:4), (18:0/22:4) and (18:0/22:6), upregulated ACSL4, LPCAT3, and ALOX15 expression level. *In vivo*, IHC revealed significant ALOX15, ACSL4, and LPCAT3 upregulated expression in SJS/TEN skin lesions. Therefore, lipid metabolism can regulate keratinocyte ferroptosis.

*SLC7A11* is the target gene for *miR-375-3*p 20. In this study, we characterized* FSP1* and *FTH1* as novel *miR-375-3p* targets in keratinocyte. FSP1 was initially identified as an agent that counteracts ferroptosis 9-10. These investigations implied that FSP1 may serve as a therapeutic target for personalized cancer therapy and suggested that changes to FSP1 expression are clinically significant. Our results confirmed that *miR-375-3p*-mediated keratinocyte ferroptosis was partially mitigated in FSP1-overexpressing cells. These results implied that boosting FSP1 activity may be a useful therapy for SJS/TEN.

FTH1 was another novel *miR-375-3p* target in keratinocyte. FTH1 is essential for maintaining iron homeostasis 38. Our study discovered that TEN Exo stimulation increased free iron and NCOA4 levels but decreased FTH1 expression. FTH1 is involved in various disease signaling pathways. FTH1 silencing in the intestines of mice can lead to iron overabsorption and promote ferroptosis 39. The antioxidant effects of epicatechin are mediated by increased FTH1 expression 40. Our studies are consistent with previous studies described above and revealed that *miR-375-3p*-mediated keratinocyte ferroptosis was partially counteracted in FTH1-overexpressing cells. In summary, FTH1 may be a promising target and therapeutically beneficial in SJS/TEN pathophysiology. Determining whether treatments that boost FSP1 and FTH1 activity are useful as therapies for SJS/TEN driven by ferroptosis is a worthy investigation. Therefore, both *miR-375-3p-*FSP1-CoQ10-NAD(P)H and *miR-375-3p*-FTH1-NCOA4 pathway play critical roles in the keratinocyte ferroptosis of SJS/TEN patients. Determining the predominant pathway in the pathogenesis of SJS/TEN is highly challenging. Additionally, dihydroorotate dehydrogenase (DHODH)/CoQH2 and GTP cyclohydrolase 1 (GCH1)/tetrahydrobiopterin (BH4) are also important defense systems to suppress ferroptosis. Moreover, exogenous monounsaturated fatty acids (MUFAs) and endosomal Sorting Complex Required for Transport-III (ESCRT-III) can act as defense pathway against ferroptosis. We would conduct a more detailed investigation into the mechanisms of these defense systems in future research.

With the deepening of research into the ferroptotic mechanism, many specific ferroptosis inhibitors have been identified. Ferrostatin-1 inhibited cell death in models of Huntington's disease 41. Importantly, patients with SJS/TEN administered 300 mg/kg NAC experienced a reversal in the evolving SJS/TEN process 30. These findings align with previous results demonstrating that lipid antioxidants significantly inhibited keratinocyte ferroptosis after TEN Exo treatment. Therefore, ferroptosis provides a novel perspective on disease management. Blocking ferroptosis may be a novel promising therapeutic strategy for SJS/TEN.

Apoptosis and ferroptosis represent distinct mechanisms of cell death. Apoptosis, a noninflammatory programmed form of cell death, can be triggered by two distinct pathways, namely, the intrinsic (also called BCL-2-regulated) pathway and the death receptor pathways 19. In our previous study, plasma exosomal *miR-375-3p* entered keratinocyte, substantially downregulated XIAP expression, and ultimately induced intrinsic keratinocyte apoptosis in patients with SJS/TEN 19. In contrast to apoptosis, ferroptosis is a ROS-dependent form of cell death associated with lipid peroxidation. In this study, we demonstrated plasma exosomal *miR-375-3p* entered keratinocyte, inducing the CoQ-dependent and ferritinophagy-mediated ferroptosis pathway in patients with SJS/TEN. Therefore, we concluded that the overexpression of *miR-375-3p* can promote apoptosis by reducing the expression of XIAP and increase ferroptosis by downregulating both FTH1 and FSP1 expression in keratinocyte.

In our previous and present studies, we have highlighted the importance of apoptosis and ferroptosis in the pathogenesis of SJS/TEN patients. Our findings indicate that blocking caspase activity with zVAD, the ferroptosis inhibitor ferrostatin-1 (Fer-1) or the inhibition of *miR-375-3*p does not completely inhibit keratinocyte death, suggesting a collaborative relationship between these two modes of cell death in SJS/TEN patients, along with potential involvement of other cell death modes. Moreover, *miR-375-3p* is a widely and functionally active noncoding RNA molecule. *MiR-375-3p* can not only triggers the ferroptosis but also promotes the apoptosis in cells 19-20. Further comprehensive investigation is necessary to establish a temporal sequence and identify potential regulatory factors governing the occurrence of apoptosis and ferroptosis in the context of SJS/TEN development.

The research presented here is subject to certain limitations. One limitation is the lack of an SJS/TEN animal model, it is essential and interesting to study SJS/TEN-related keratinocyte lipid metabolism dysregulation* in vivo* after treatment with *miR-375-3p* inhibitor and FSP1 or FTH1 antagonists. However, the initiation of this process is hindered by various factors, including difficulties in obtaining ethical consent from patients, challenges in excising skin lesions from patients who have recovered from SJS/TEN, the ambiguity of causative drugs, and the intricacies of replicating human SJS/TEN progression *in vivo*. Another limitation of this work relates to collecting samples at an early disease stage, which would be essential to fully understand pathogenic mechanisms. This work would be challenging because of the diseases' rarity, the difficulty in diagnosing them at early stage, and their rapid progression, the patient was already in an advanced stage upon admission to the hospital. The third limitation is the small sample size of patients in the transcriptome and lipidomics analysis. Owning to the usefulness of human leukocyte antigen (HLA) screening before administering specific drugs, the incidence of SJS/TEN has significantly decreased in recent years. Therefore, acquiring an adequate number of patient samples proves challenging, resulting in a significant decrease in sample size for transcriptome and lipidomics analyses. We are currently focusing on collaborating with several dermatology departments to expand our sample collection. Developing a human epidermal keratinocyte SJS/TEN animal model is crucial and warrants attention in future research endeavors.

In conclusion, therapeutic strategies targeting lipid metabolism, CoQ-dependent pathway and ferritinophagy-mediated ferroptosis may effectively prevent keratinocyte death in SJS/TEN.

## Supplementary Material

Supplementary figures and tables.
