# Supplementary material for: Exosomal miR-375-3p mediated lipid metabolism, ferritinophagy and CoQ-dependent pathway contributes to the ferroptosis of keratinocyte in SJS/TEN
Source: Int J Biol Sci. 2025 Jan 20;21(3):1275–93. doi: 10.7150/ijbs.98592 (PMC11781181; doi:10.7150/ijbs.98592)
Supplement: Supplementary file 1 — Supplementary figures and tables. [file ijbsv21p1275s1.pdf]

Figure S1. Ferroptosis-related protein expression *in vivo*. (**A**) 8-OHdG and 4-HNE immunostaining in MPE patients. (**B**) Rabbit IgG isotype control immunostaining. *Bar* = 100 μm.

Figure S2. Ferroptosis-related protein expression *in vivo*. TFRC, STEAP3, and DMT1 immunostaining. *Bar* = 100 μm.

Figure S3. Characterization of plasma-derived exosomes. (**A**) Electron micrographs. *Bar* = 100 μm. (**B**) Western blotting confirmed the presence of exosome marker proteins. Positive control, CD9 and TSG101: HEPG2 cell lines; positive control, CD81: 293T cell lines. (**C**) Nano-Flow Cytometry (NanoFCM) confirmed the marker proteins CD9, CD63 and CD81 in plasma-derived exosome. (**D**) Nanoparticle tracking analysis of plasma exosome size distribution. Con Exo, plasma-derived exosome from healthy individuals; EM Exo, plasma-derived exosome from patients with EM; and TEN Exo, plasma-derived exosome from patients with SJS/TEN.

Figure S4. Plasma-derived exosome induced ferroptosis in HaCaT cells. (**A**) Plasma-derived exosomes stained with DiI red membrane dye. Nuclei counterstained with Hoechst (blue). *Bar* = 100 μm. (**B**) The protein expression levels of FSP1, ACSL4, PTGS2, TFRC, and GPX4 in exosomes were analyzed by western blotting. (**C**) Lipid peroxidation accumulation. *Bar* = 100 μm. $^{*}P < 0.05$, $^{**}P < 0.01$, $^{***}P < 0.001$.

Figure S5. Ferroptosis-related protein expression *in vivo*. ALOX15, LPCAT3, and ACSL4 immunostaining. *Bar* = 100 μm.

Figure S6. NCOA4-FTH1 interaction contributes to the ferroptosis of

keratinocyte stimulated by TEN Exo. (**A**) NCOA4 and FTH1 protein expression levels were analyzed by western blotting after treatment with Con Exo, EM Exo, and TEN Exo (100 μg/mL). (**B**) The protein levels of FTH1 and NCOA4 in HaCaT cells stimulated with TEN Exo for indicated times. (**C**) The intracellular iron level in HaCaT cells stimulated by TEN Exo or Con Exo for indicated times. (**D**) The intracellular iron level in HaCaT cells stimulated by TEN Exo with or without the iron chelator DFO. (**E**) The expression levels of autophagy biomarkers were analyzed by western blotting after treatment with TEN Exo (100 μg/mL) and 3-MA. The data are reported as the means ± SEMs of three independent experiments. *Bar* = 100 μm. *ns*, not significant. **P* < 0.05, ***P* < 0.01, ****P* < 0.001.

Figure S7. NCOA4-mediated ferritinophagy contributes to the keratinocyte ferroptosis treated by TEN Exo. (**A**) NCOA4 protein expression. (**B-C**) The mRNA and protein levels of PTGS2 and ALSC4 in human primary keratinocytes stimulated with TEN Exo after *NCOA4* siRNA transfection. (**D-H**) The protein levels of NCOA4 and FTH1 (**D**), oxLDL levels (**E**), intracellular ROS (**F**), MDA levels (**G**), lipid peroxidation accumulation (**H**) in human primary keratinocytes stimulated with TEN Exo after *NCOA4* siRNA transfection. The data are reported as the means ± SEMs of three independent experiments. *Bar* = 100 μm. *ns*, not significant. **P* < 0.05, ***P* < 0.01, ****P* < 0.001.

Figure S8. Ferroptosis-related protein expression *in vivo*. FSP1, NCOA4, and FTH1 immunostaining. *Bar* = 100 μm.

Figure S9. *miR-375-3p* expression in patients with SJS/TEN. RNAscope *in situ*

miR-375-3p detection. *Bar* = 200 μm.

Figure S10. *miR-375-3p*-Exo-Fect exosome internalization and ferroptosis promotion *in vitro*. (**A**) *miR-375-3p* exosome absorbed by HaCaT cells. *Bar* = 100 μm. (**B-H**) HaCaT cells were incubated with *miR-375-3p*-loaded TEN Exo for 48 h. (**B**) Relative *miR-375-3p* expression was detected by qRT–PCR. (**C**) MDA accumulation. (**D**) NADPH levels. (**E**) Lipid peroxidation accumulation. (**F**) Intracellular ROS levels. (**G**) oxLDL levels. (**H**) Ferroptosis protein expression were analyzed by western blotting. *Bar* = 100 μm. The data are reported as the means ± SEMs of three independent experiments. **P* < 0.05, ***P* < 0.01, ****P* < 0.001.

Figure S11. *miR-375-3p*-induced the ferroptosis of keratinocyte. (**A**) qRT–PCR confirming changes in *miR-375-3p* in HaCaT cells transfected with a negative control mimic (NC mimic), an NC inhibitor, a *miR-375-3p* mimic, or a *miR-375-3p* inhibitor and normalized to *miR-139* expression. (**B-L**) HaCaT cells transfected with an NC mimic, an NC inhibitor, a *miR-375-3p* mimic, or a *miR-375-3p* inhibitor. (**B**) MDA accumulation. (**C**) Fe^{2+} accumulation. (**D**) LIP levels. (**E**) Intracellular ROS levels. (**F**) Lipid peroxidation. (**G**) The intracellular iron level. (**H-J**) NAD(P)H and CoQ10 levels. (**K**) oxLDL levels. (**L**) ACSL4 and PTGS2 protein expression were analyzed by western blotting. The data are reported as the means ± SEMs of three independent experiments. *Bar* = 100 μm. ***P* < 0.01, ****P* < 0.001.

Figure S12. Analysis of *miR-375-3p* binding sites in the *GCH1* and *DHODH* 3′-UTR or GPX4 5′-UTR. (**A**) Schematic diagram showing putative *miR-375-3p* binding sites in the 5'-UTR of human *GPX4* and HaCaT cells transfected with

wild-type or mutant *GPX4* 5'-UTR luciferase and negative control mimic or *miR-375-3p* mimic. (**B-C**) Schematic diagram showing putative *miR-375-3p* binding sites in the 3'-UTR of human *GCH1* or *DHODH* and HaCaT cells transfected with wild-type or mutant *GCH1* or *DHODH* 3'-UTR luciferase and negative control mimic or *miR-375-3p* mimic. *ns*, not significant, ***$P < 0.001$.

Figure S13. *FSP1* knockdown promoted ferroptosis of human primary keratinocytes. (**A-B**) FSP1 mRNA and protein expression in human primary keratinocytes. (**C-H**) Human primary keratinocytes transfected with *FSP1*-siRNA for 48 h. (**C**) Cell viability. (**D**) MDA level. (**E-F**) NAD(P)H levels. (**G**) CoQ10 levels. (**H**) Lipid peroxidation accumulation. (**I**) Intracellular ROS levels. *Bar* = 100 μm. The data are reported as the means ± SEMs of three independent experiments. *ns*, not significant. *$P < 0.05$, **$P < 0.01$, ***$P < 0.001$.

Figure S14. *FTH1* knockdown promoted ferroptosis of human primary keratinocytes. (**A-B**) FTH1 mRNA and protein expression in human primary keratinocytes. (**C-K**) Human primary keratinocytes transfected with *FTH1*-siRNA for 48 h. (**C**) The intracellular iron level. (**D**) MDA level. (**E**) Cell viability. (**F**) LIP levels. (**G**) Intracellular ROS. (**H**) oxLDL levels. (**I**) Lipid peroxidation accumulation. *Bar* = 100 μm, (**J-K**) NCOA4, TFRC, and LC3B mRNA and protein expression. The data are reported as the means ± SEMs of three independent experiments. *ns*, not significant. *$P < 0.05$, **$P < 0.01$, ***$P < 0.001$.

Figure S15. TNF-α could not mediate the ferroptosis of human primary keratinocytes. (**A-E**) Human primary keratinocytes were treated with TNF-α for 48 h.

(**A**) The expression levels of ferroptosis biomarkers in human primary keratinocytes were analyzed by western blotting. (**B**) Intracellular ROS level. (**C**) MDA level. (**D**) The intracellular iron level. (**E**) Lipid peroxidation accumulation. *Bar* = 100 μm. The data are reported as the means ± SEMs of three independent experiments. *ns*, not significant.

Figure S16. XIAP could not mediate the ferroptosis of human primary keratinocytes. (**A-E**) Human primary keratinocytes were transfected with *XIAP*-siRNA for 48 h. (**A**) The expression levels of ferroptosis biomarkers in human primary keratinocytes were analyzed by western blotting. (**B**) Intracellular ROS level. (**C**) MDA level. (**D**) The intracellular iron level. (**E**) Lipid peroxidation accumulation. *Bar* = 100 μm. The data are reported as the means ± SEMs of three independent experiments. *ns*, not significant.

Figure S17. Exosomal *miR-375-3p* levels in SJS/TEN patients are positively correlated with biomarkers of ferroptosis. Exosomal *miR-375-3p* levels were positively correlated with Fe^{2+} and MDA accumulation and negatively correlated with CoQ10 levels in SJS/TEN patients.

Table 1. Clinical characteristics for SJS/TEN.

Num.	Age	C-P(ng/ml)	Cause	Glucose(mmol/l)	SCORTEN	BSA(%)	CRP(mg/l)	DBIL(u/l)	Agender
1	13	2.744	carbamazepine	6.8	2	70	37.7	19.3	Male
2	55	0.398	lamotrigine	13.13	1	6	95.2	11.9	Female
3	14	0.358	carbamazepine	6.7	1	45	30.15	8.7	Female
4	26	2.427	amoxicillin	5.0	1	60	30.40	9.0	Male
5	29	1.627	unknown	6.1	0	5	10.5	7.0	Female
6	43	3.142	carbamazepine	4.76	2	75	34.3	4.6	Male
7	63	2.167	penicillin	5.50	1	1	24.5	9.0	Female
8	66	0.298	unknown	10.23	2	1	7.96	9.0	Female
9	16	3.444	sulfonamides	5.69	1	9	31	6.8	Male
10	24	3.713	lincomycin	3.30	1	2	33.6	5.4	Female
11	16	2.799	antondine	3.76	1	38	17.6	3.1	Female
12	58	3.825	unknown	4.44	1	5	9.8	4.4	Female
13	7	3.799	lamotrigine	3.53	0	6	67.15	7.3	Male
14	47	0.195	unknown	21.80	4	85	153	39.0	Female
15	35	0.305	lamotrigine	4.00	2	76	5.14	13.4	Male
16	50	3.969	carbamazepine	6.60	2	34	85.45	15.4	Female
17	77	2.275	lamotrigine	4.21	1	2	87.3	3.5	Male
18	52	3.836	sulfonamides	5.74	1	9	37.1	11.6	Female
19	26	0.215	acetaminophen	6.40	2	41	35.5	11.2	Male
20	50	2.111	carbamazepine	8.20	2	70	85.6	2.7	Female
21	34	2.298	amoxicillin	5.50	0	7	12.1	5.2	Female
22	30	4.057	amoxicillin	7.70	1	60	12.0	3.0	Male
23	67	0.291	brinzolamide	9.10	2	28	116	12.0	Female
24	6	2.937	unknown	2.90	0	30	7.8	10.0	Female
25	16	2.618	lincomycin	7.50	0	5	10.7	4.5	Female
26	15	0.421	lincomycin	5.20	1	35	47.7	3.9	Male
27	44	0.311	unknown	8.60	2	19	9.85	13.4	Female
28	53	3.877	carbamazepine	8.20	2	30	148	6.2	Female
29	19	2.368	carbamazepine	4.60	0	2	11.6	5.1	Female
30	34	3.141	sulfonamides	5.50	0	6	1.29	7.5	Male
31	36	1.726	carbamazepine	7.69	2	70	72.9	10.2	Female
32	55	0.338	roxithromycin	13.60	4	90	265	29.0	Female
33	12	3.125	unknown	5.40	0	3	6.6	6.8	Male
34	16	1.173	carbamazepine	5.10	1	40	15.5	8.4	Female
35	63	1.284	penicillin	11.60	2	60	116	28.0	Male

Table 2. Institutional Review Board approval used in the studies

Approval Form of IEC, First Affiliated Hospital of Fourth Military Medical University

Ethics Committee Approval No.: No. KY20172030-1

<table>
<tr><td>Project</td><td colspan="5">Clinical Study of the Establishment of a Biological Sample Bank</td></tr>
<tr><td rowspan="2">Department</td><td rowspan="2">Dermatology</td><td>Principal Investigator</td><td>Wang Gang</td><td>Title</td><td>Chief physician/professor</td></tr>
<tr><td>Director of Department</td><td>Wang Gang</td><td>Title</td><td>Chief physician/professor</td></tr>
<tr><td>Purpose</td><td colspan="5">To establish a biological sample bank for inflammatory skin diseases such as psoriasis and vitiligo, autoimmune bullous diseases, skin tumors such as melanoma, allergic skin diseases, and hereditary skin diseases that meets international standards</td></tr>
<tr><td>Forms to be handed</td><td colspan="5">1. Submission letter; 2. Reply to ethics review opinions; 3. Clinical study protocol (version number: V2.0, version date: June 16, 2017); 4. Informed consent form (version number: V2.0, version date: June 16, 2017); 5. Case report form V2.0, version date: June 16, 2017; 6. Application form for review work</td></tr>
<tr><td>Category</td><td colspan="5">Initial Review ☐ Re-review ■ Follow-up Review ☐</td></tr>
<tr><td>Date</td><td>June 29, 2017</td><td>Locus</td><td colspan="3">Conference Room, 20th Floor, Digestive Disease Hospital, First Affiliated Hospital of Fourth Military Medical University</td></tr>
<tr><td>Way</td><td colspan="5">Meeting Review ☐ Emergency Meeting Review ☐ Fast Review ■</td></tr>
<tr><td>Determination</td><td colspan="5">Approved ■; To be approved after necessary amendments ☐; To be re-reviewed after necessary amendments ☐ ; Disapproved ☐ ; Suspension or termination of the approved trial ☐</td></tr>
<tr><td>Annual/Periodic Continuing Review</td><td colspan="5">Yes ■ No ☐</td></tr>
<tr><td>Follow-up review frequency (from the date of approval of the trial)</td><td colspan="5">3 months ☐ 6 months ☐ 9 months ☐ 12 months ■ None ☐</td></tr>
<tr><td colspan="6">Comments:
After review by this ethics committee, it is approved to carry out this study according to the approved clinical study protocol and informed consent form. The investigator and sponsor are required to strictly conform to GCP principles, abide by relevant national laws and regulations, follow the protocol approved by the ethics committee in conducting the clinical study, fully implement the informed consent form, and effectively protect the rights and safety of study subjects. Before the study starts, the investigator should complete the clinical trial registration. Matters requiring attention in the study process:
1. If the principal investigator changes during the study process, any amendment to the clinical study protocol, informed consent form, and recruitment materials, etc. may only be implemented after review and approval by the ethics committee.
2. The annual/periodic review frequency specified by the ethics committee should be followed, and the investigator should submit a study progress report one month before the deadline. A summary report on the research progress of each study site should be submitted to the ethics committee of the leading site; in the event that there is any situation that may significantly affect the implementation of the trial or increase the risk to the subjects, the investigator should submit a written report to the ethics committee in a timely manner.</td></tr>
</table>

3. The following situations should be reported in time during the trial: (1) Any serious adverse events should be reported within 24 hours; (2) In the event that the study is not carried out in compliance with the protocol, e.g., enrolling subjects who do not meet the inclusion criteria or meet the exclusion criteria, not withdrawing subjects who meet the requirements for discontinuation of the trial, giving wrong treatment or dosage, and giving concomitant medications prohibited by the protocol; or is in violation of GCP principles, e.g., potentially having adverse effects on the rights/health of the subjects and the scientificity of the research, etc., the investigator should submit a protocol violation report; and (3) In the event that the investigator discontinues or terminates the clinical study in advance, the investigator should submit the discontinuation/termination report in time.<br>4. Upon completion of the clinical study, the investigator should submit the study summary report.<br>Ethical Committee for Clinical Trials of Drugs in the First Affiliated Hospital of the Fourth Military Medical University<br>Signature of Chairman of Ethics Committee: TianWen Gao<br>June 29, 2017<br>[Seal] Ethics Committee of Drug Clinical Trials in First Affiliated Hospital of Fourth Military Medical University
Statement: The responsibilities, personnel composition, operating procedures, and records of this ethics committee follow ICH-GCP/China GCP and applicable Chinese laws and regulations. All present members of the ethics committee are in their effective term of office and will keep confidential the clinical study materials reviewed and the results of the ethics committee meeting and related contents, and they have no conflict of interest in this study project. This approval document is valid for three years and will be invalidated automatically upon expiration.

Address: 127 Changle West Road, Xi’an Post code: 710032 Tel: 029-84771794

Table 3. Sequence information for siRNA used in the studies Gene

hFTH1	Sense-1: CCUGUCCAUGUCUUACUACUUTT
	Antisense-1: AAGUAGUAAGACAUGGACAGGTT
	Sense-2: CCUUCAGGAUAUCAAGAAATT
	Antisense-2: UUUCUUGAUAUCCUGAAGGTT
	Sense-3: AGAUCAACCUGGAGCUCUATT
	Antisense-3: UAGAGCUCCAGGUUGAUCUTT
hFSP1	Sense-1: CCAAAUCAGUGGCUUCUAUdTdT
	Antisense-1: AUAGAAGCCACUGAUUUGGdTdT
	Sense-2: GCACCGGCAUCAAGAUCAAdTdT
	Antisense-2: UUGAUCUUGAUGCCGGUGCdTdT
	Sense-3: GCUGCCUCUCAAUGAGUAUdTdT
	Antisense-3: AUACUCAUUGAGAGGCAGCdTdT
hNCOA4	Sense-1: UCAGCAGCUCUACUCGUUAUUdTdT
	Antisense-1: AAUAACGAGUAGAGCUGCUGAdTdT
	Sense-2: UGAACAGGUGGACCUUAUUUAdTdT
	Antisense-2: UAAAUAAGGUCCACCUGUUCAdTdT
	Sense-3: CUCUUAUUCCAGUCCUAUAAUdTdT
	Antisense-3: AUUAUAGGACUGGAAUAAGAGdTdT

Table 4. Sequence information for RT-PCR primers used in the studies

Gene	**Primers (5′→3′)**
GPX4	F: GCACATGGTTAACCTGGACA
	R: CTGCTTCCCGAACTGGTTAC
PTGS2	F:CTGCGCCTTTTCAAGGATGG
	R: GGGGATACACCTCTCCACCA
ACSL4	F:CCCCACCCACTCCCATCT
	R: GAATTAGCAGCACCCAACCTTA
FSP1	F: GGGTTCGCCAAAAAGACATTCATTT
	R: CCAACTTGCTCATTCCTACCCTTTTCTG
TFRC	F: CTCCAGAGCTGCTGCAGAAAAGC
	R: CTCCCTGAATAGTCCAAGTAGC
ALOX15	F: TGGCTGCCCCGCTGGTCATG
	R: CTCCCTGAATAGTCCAAGTAGC
LPCAT3	F: ACCCCTTTGCTTTGTTTTATCG
	R: TAAGCAATTGAGAGGCCTGTAA

FTH1	F: TGTGGCGGAGCTGCTGGGTAA
	R: CGAGAGGTGGATACGGCTGCT
FASN	F: TACGTACTGGCCTACACCCAGA
	R: TGAACTGCTGCACGAAGAAGCATAT
FADS2	F: TTACAACATCACCAAATGGTCCAT
	R: GAAGGCATCCGTTGCATCTT
SCD1	F: CATAATTCCCGACGTGGCTTT
	R: AGGTTTGTAGTACCTCCTCTGGAACA
SREBP1	F: CGCAAGGCCATCGACTACAT
	R: GACTTAGGTTCTCCTGCTTGAGTTTC
LC3B	F: TGTCCGACTTATTCGAGAGCAGCA
	R: TTCACCAACAGGAAGAAGGCCTGA
NCOA4	F: GAGGTGTAGTGTGCACGGAG
	R: GACGGCTTATGCAACTGTGAA
18S	F: AGTCCCTGCCCTTTGACACA
	R: GATCCGAGGGCCTCACTAAAC

Figure S1

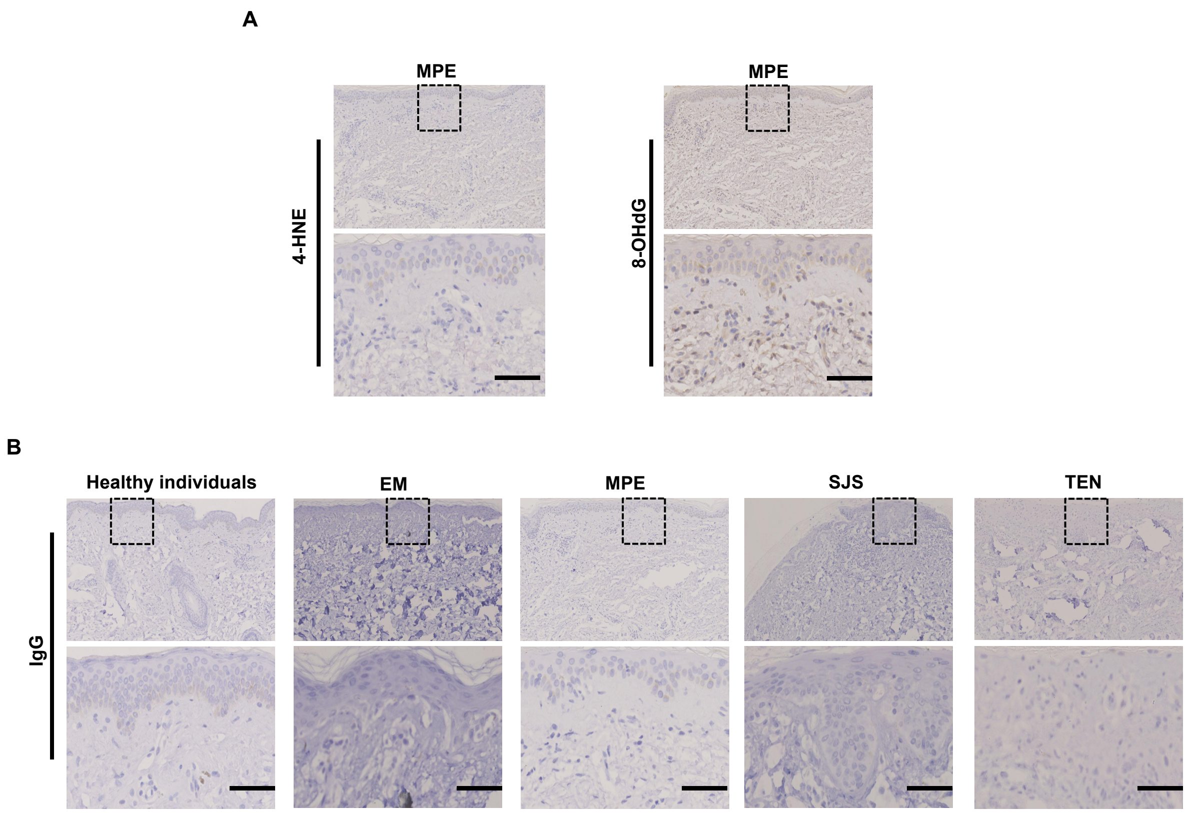

Figure S2

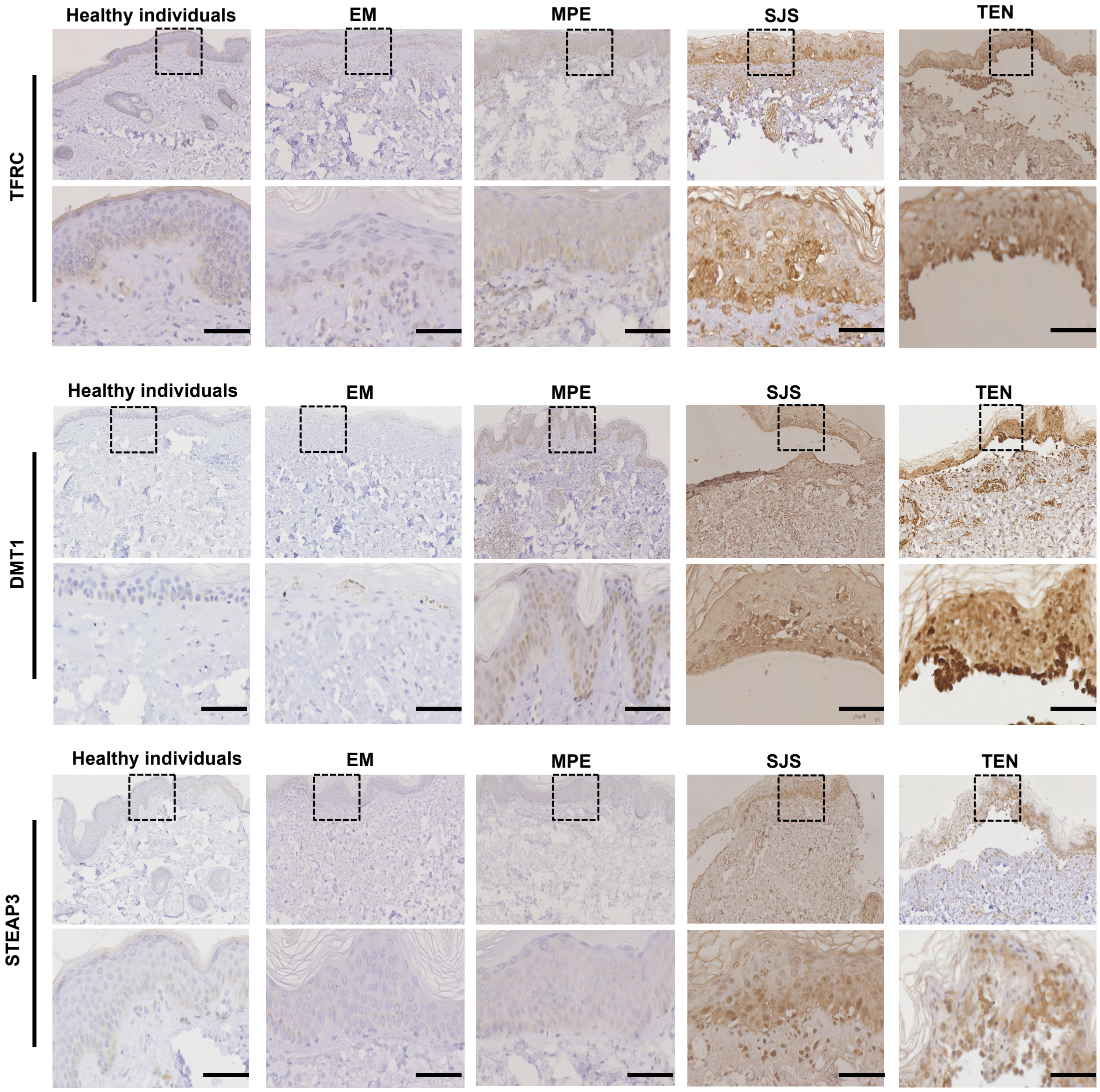

Figure S3

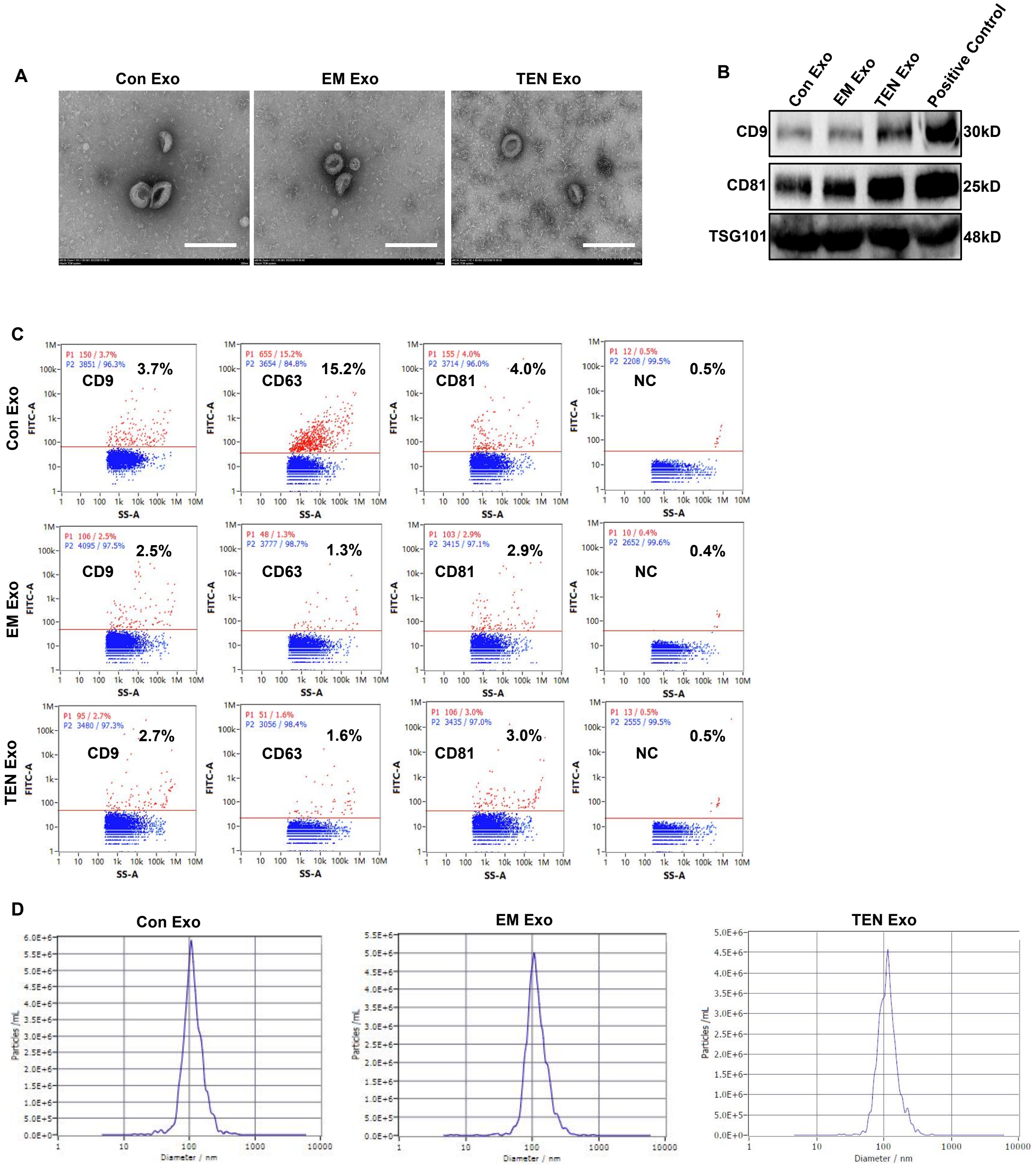
A
Con Exo
EM Exo
TEN Exo
B
Con Exo
EM Exo
TEN Exo
Positive Control
CD9
30kD
CD81
25kD
TSG101
48kD
C
Con Exo
EM Exo
TEN Exo
CD9
CD63
CD81
NC
3.7%
15.2%
4.0%
0.5%
2.5%
1.3%
2.9%
0.4%
2.7%
1.6%
3.0%
0.5%
FITC-A
SS-A
D
Con Exo
EM Exo
TEN Exo
Particles /mL
Diameter / nm

Figure S4

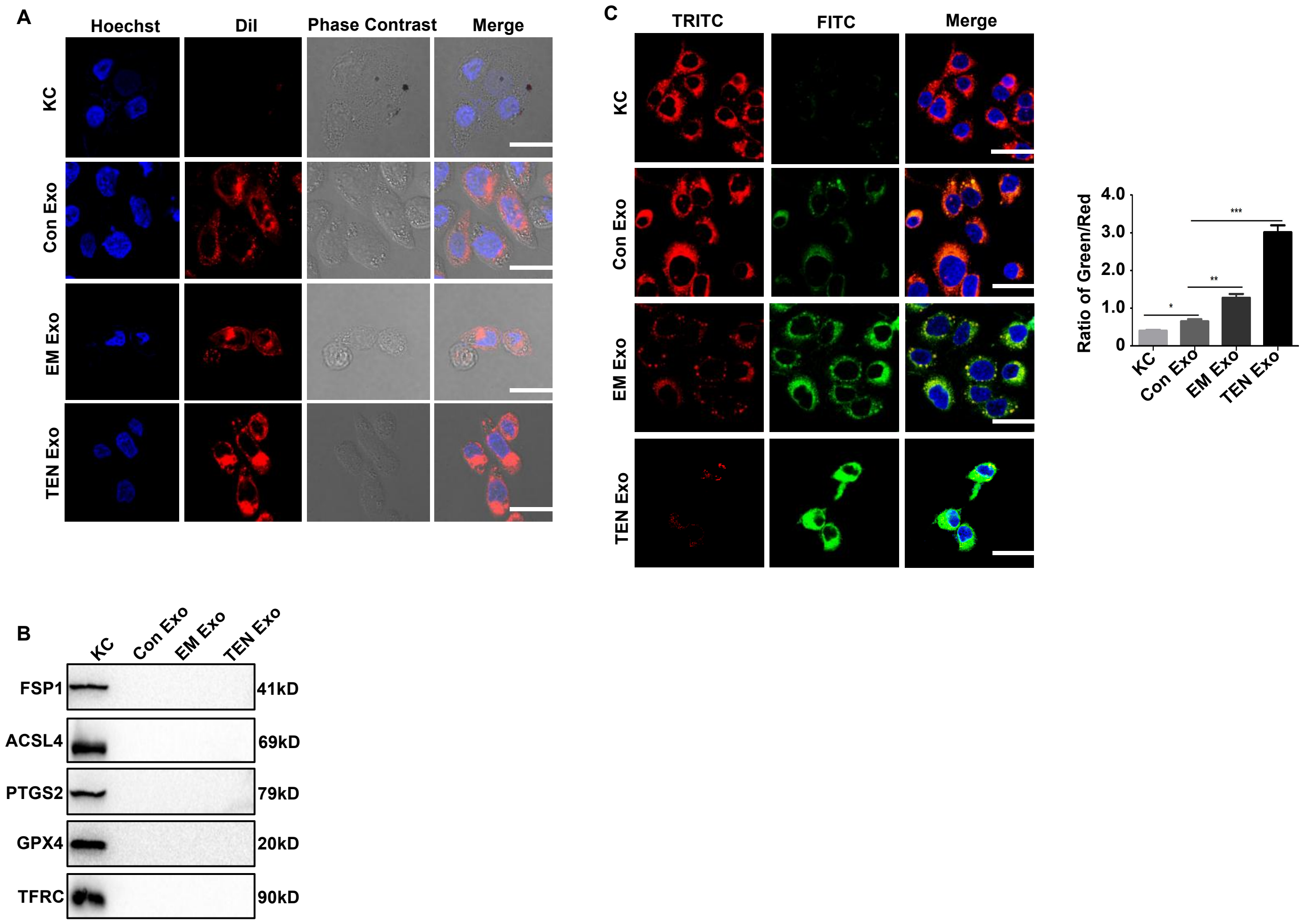
A
Hoechst
DiI
Phase Contrast
Merge
KC
Con Exo
EM Exo
TEN Exo
B
KC
Con Exo
EM Exo
TEN Exo
FSP1
41kD
ACSL4
69kD
PTGS2
79kD
GPX4
20kD
TFRC
90kD
C
TRITC
FITC
Merge
KC
Con Exo
EM Exo
TEN Exo
Ratio of Green/Red
4.0
3.0
2.0
1.0
0
*
**
***
KC
Con Exo
EM Exo
TEN Exo

Figure S5

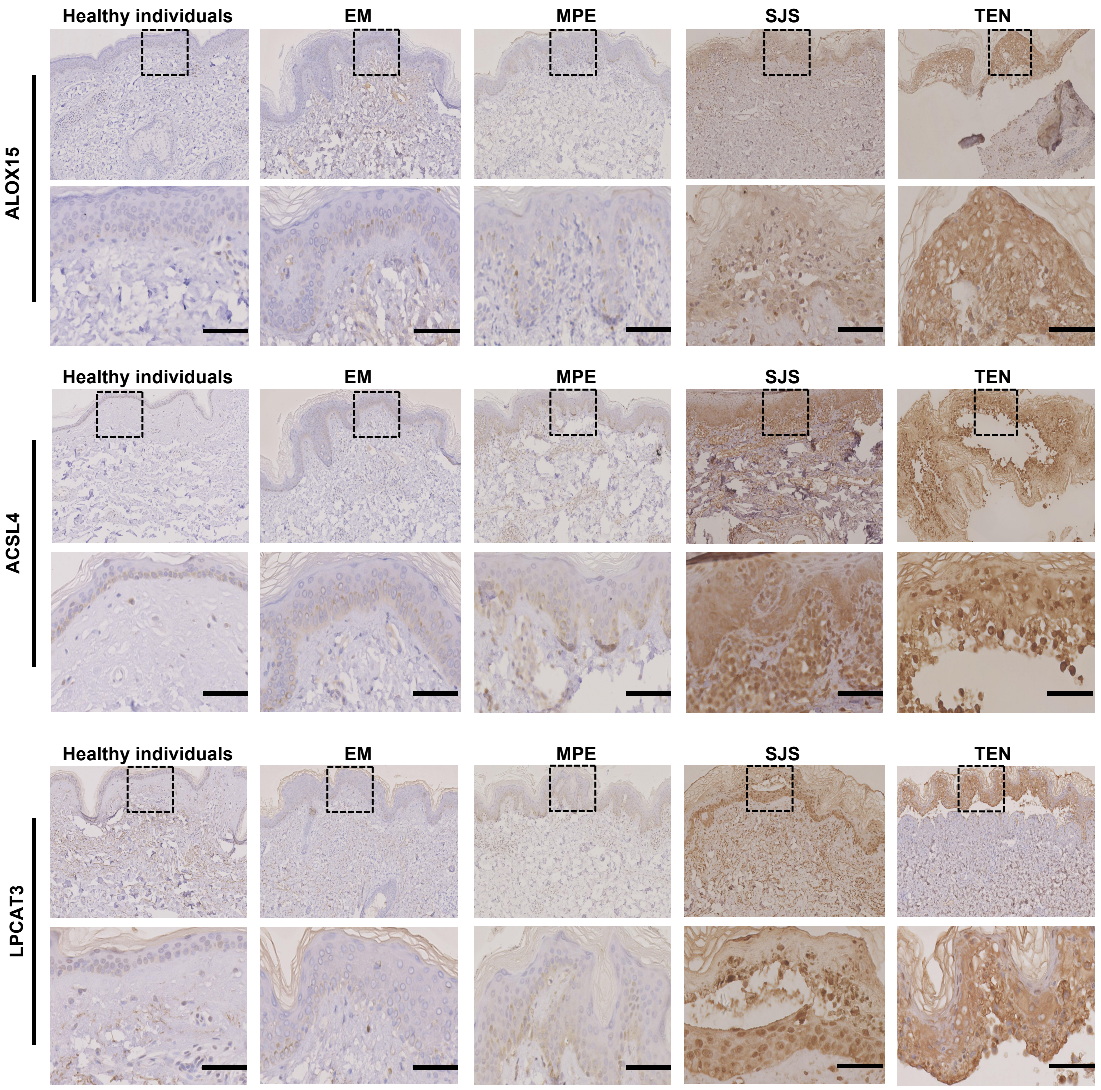

Figure S6

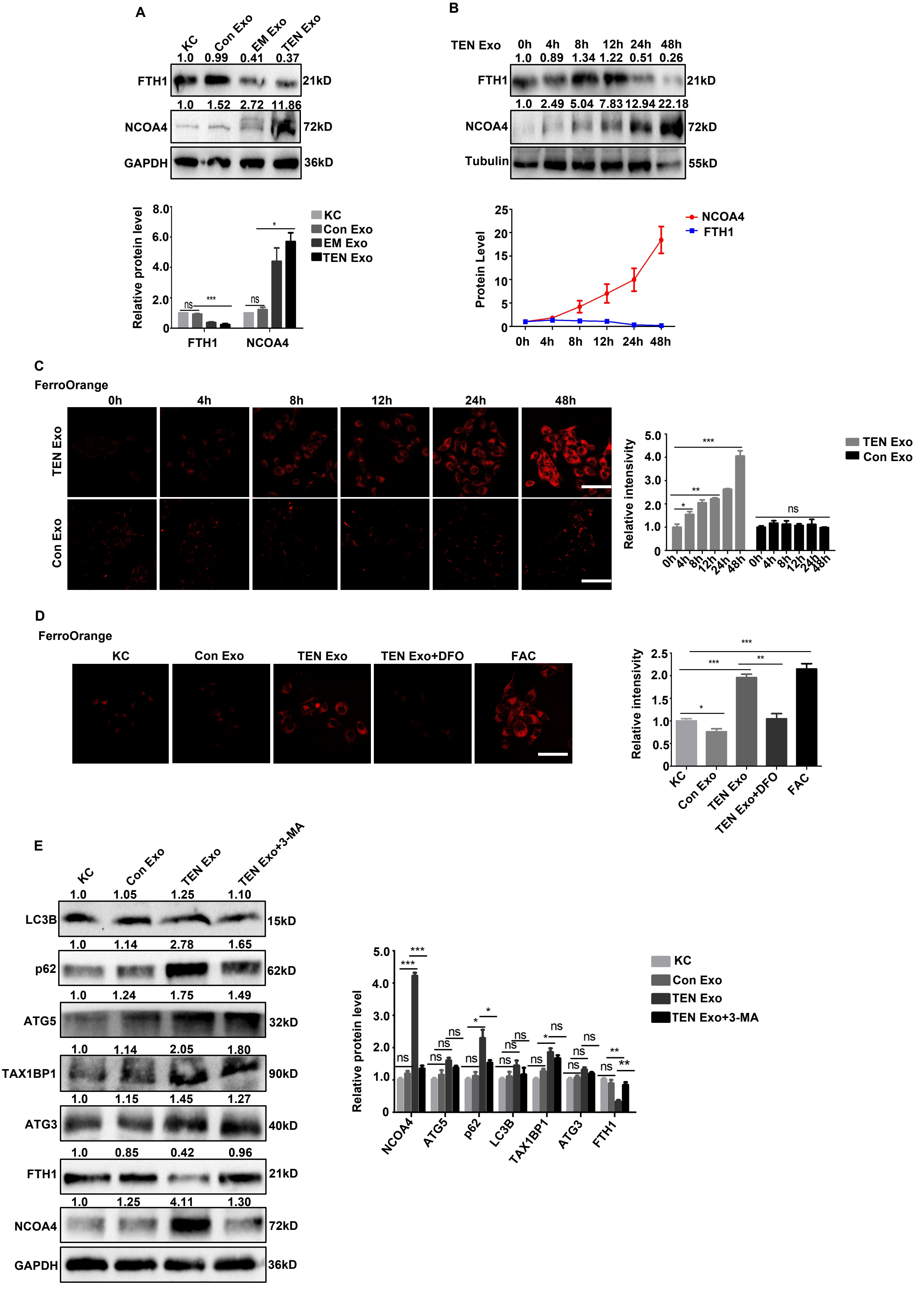
A
KC
Con Exo
EM Exo
TEN Exo
1.0 0.99 0.41 0.37
FTH1
21kD
1.0 1.52 2.72 11.86
NCOA4
72kD
GAPDH
36kD
Relative protein level
FTH1
NCOA4
KC
Con Exo
EM Exo
TEN Exo
B
TEN Exo 0h 4h 8h 12h 24h 48h
1.0 0.89 1.34 1.22 0.51 0.26
FTH1
21kD
1.0 2.49 5.04 7.83 12.94 22.18
NCOA4
72kD
Tubulin
55kD
Protein Level
NCOA4
FTH1
C
FerroOrange
0h 4h 8h 12h 24h 48h
TEN Exo
Con Exo
Relative intensivity
TEN Exo
Con Exo
D
FerroOrange
KC Con Exo TEN Exo TEN Exo+DFO FAC
Relative intensivity
E
KC
Con Exo
TEN Exo
TEN Exo+3-MA
1.0 1.05 1.25 1.10
LC3B
15kD
1.0 1.14 2.78 1.65
p62
62kD
1.0 1.24 1.75 1.49
ATG5
32kD
1.0 1.14 2.05 1.80
TAX1BP1
90kD
1.0 1.15 1.45 1.27
ATG3
40kD
1.0 0.85 0.42 0.96
FTH1
21kD
1.0 1.25 4.11 1.30
NCOA4
72kD
GAPDH
36kD
Relative protein level
NCOA4
ATG5
p62
LC3B
TAX1BP1
ATG3
FTH1
KC
Con Exo
TEN Exo
TEN Exo+3-MA

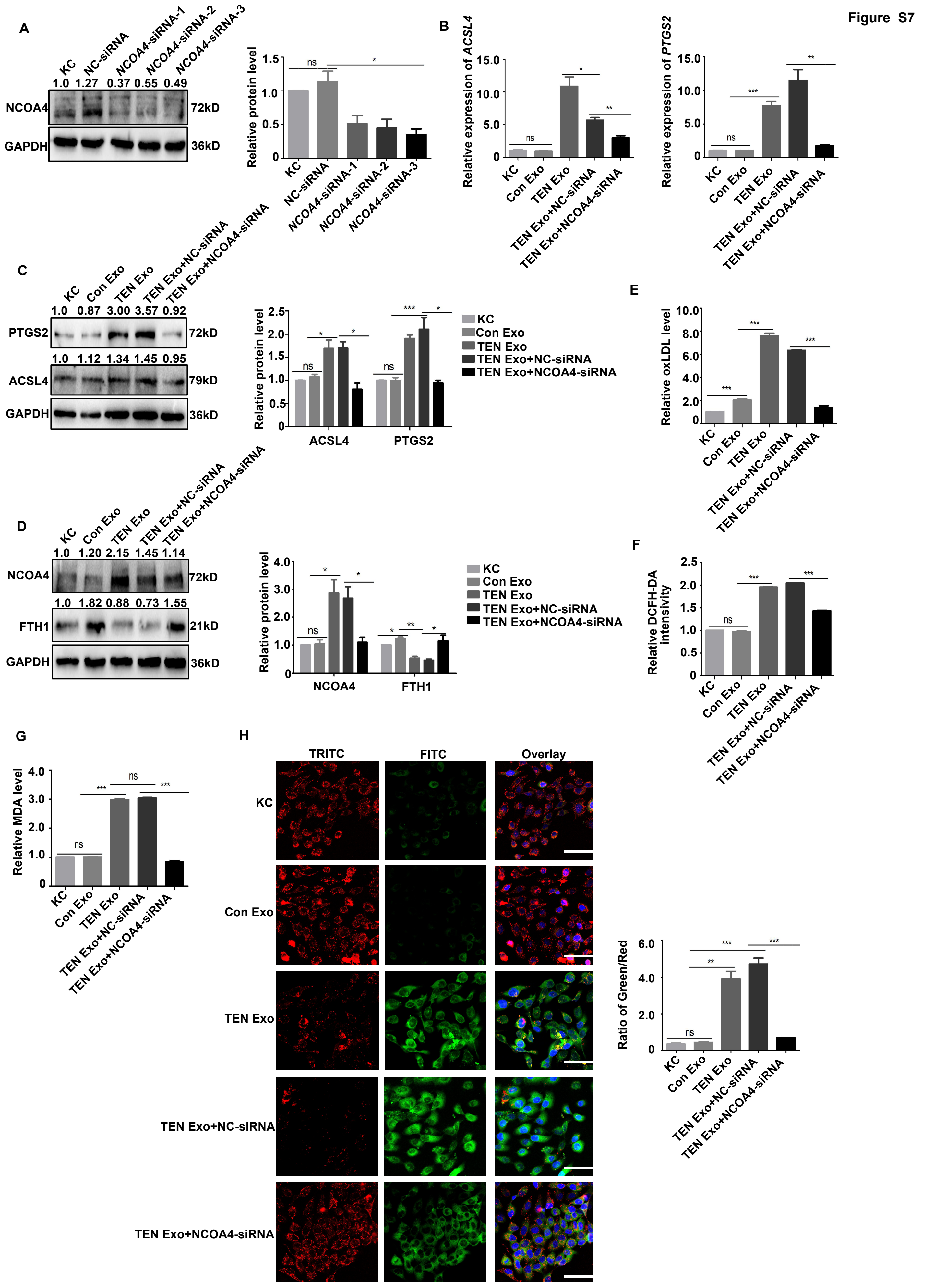

Figure S7
A
KC
NC-siRNA
NCOA4-siRNA-1
NCOA4-siRNA-2
NCOA4-siRNA-3
1.0 1.27 0.37 0.55 0.49
NCOA4
72kD
GAPDH
36kD
Relative protein level
B
Relative expression of ACSL4
Relative expression of PTGS2
KC
Con Exo
TEN Exo
TEN Exo+NC-siRNA
TEN Exo+NCOA4-siRNA
C
1.0 0.87 3.00 3.57 0.92
PTGS2
72kD
1.0 1.12 1.34 1.45 0.95
ACSL4
79kD
GAPDH
36kD
ACSL4
PTGS2
E
Relative oxLDL level
D
1.0 1.20 2.15 1.45 1.14
NCOA4
72kD
1.0 1.82 0.88 0.73 1.55
FTH1
21kD
GAPDH
36kD
NCOA4
FTH1
F
Relative DCFH-DA intensivity
G
Relative MDA level
H
TRITC
FITC
Overlay
Ratio of Green/Red

Figure S8

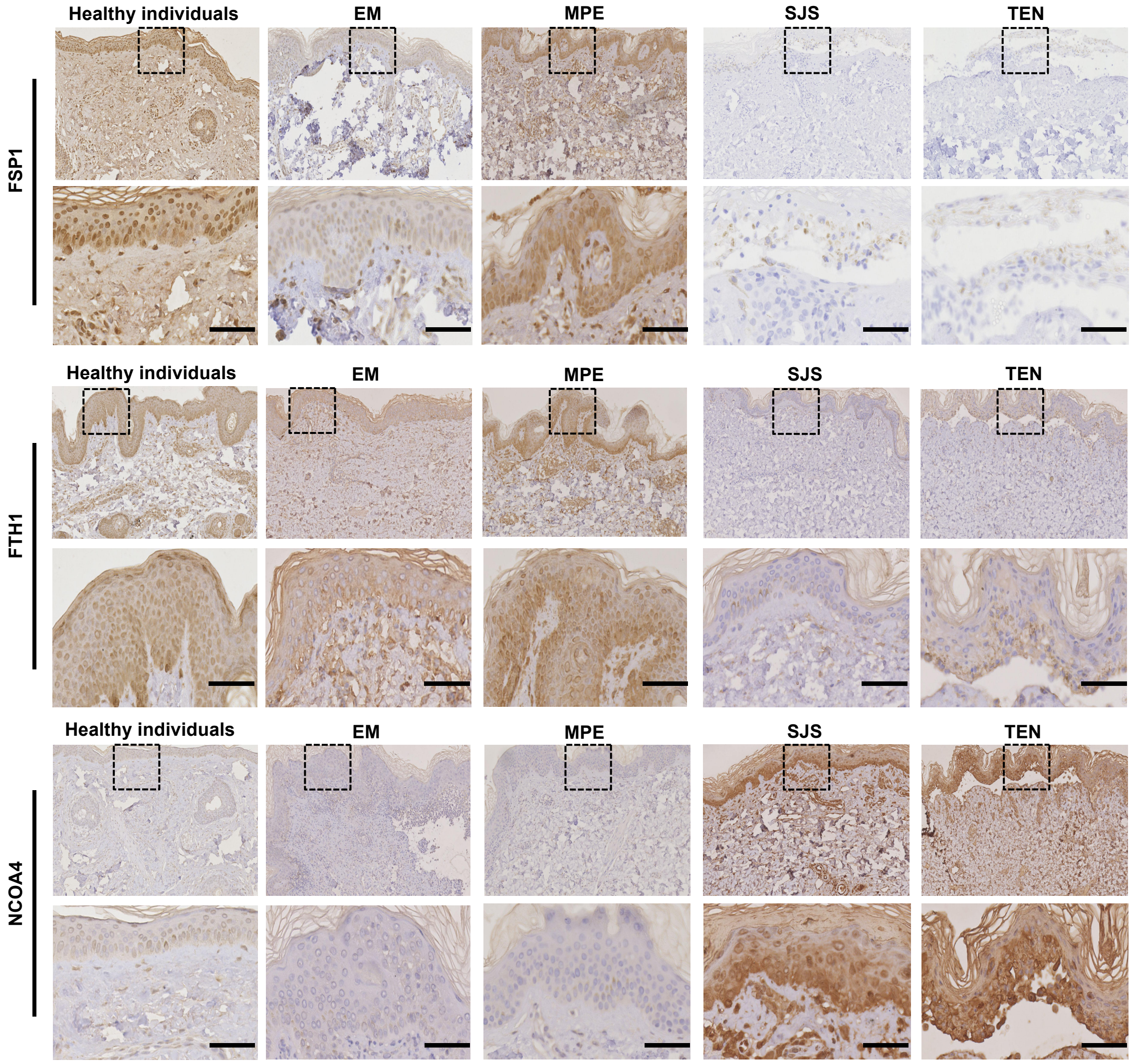

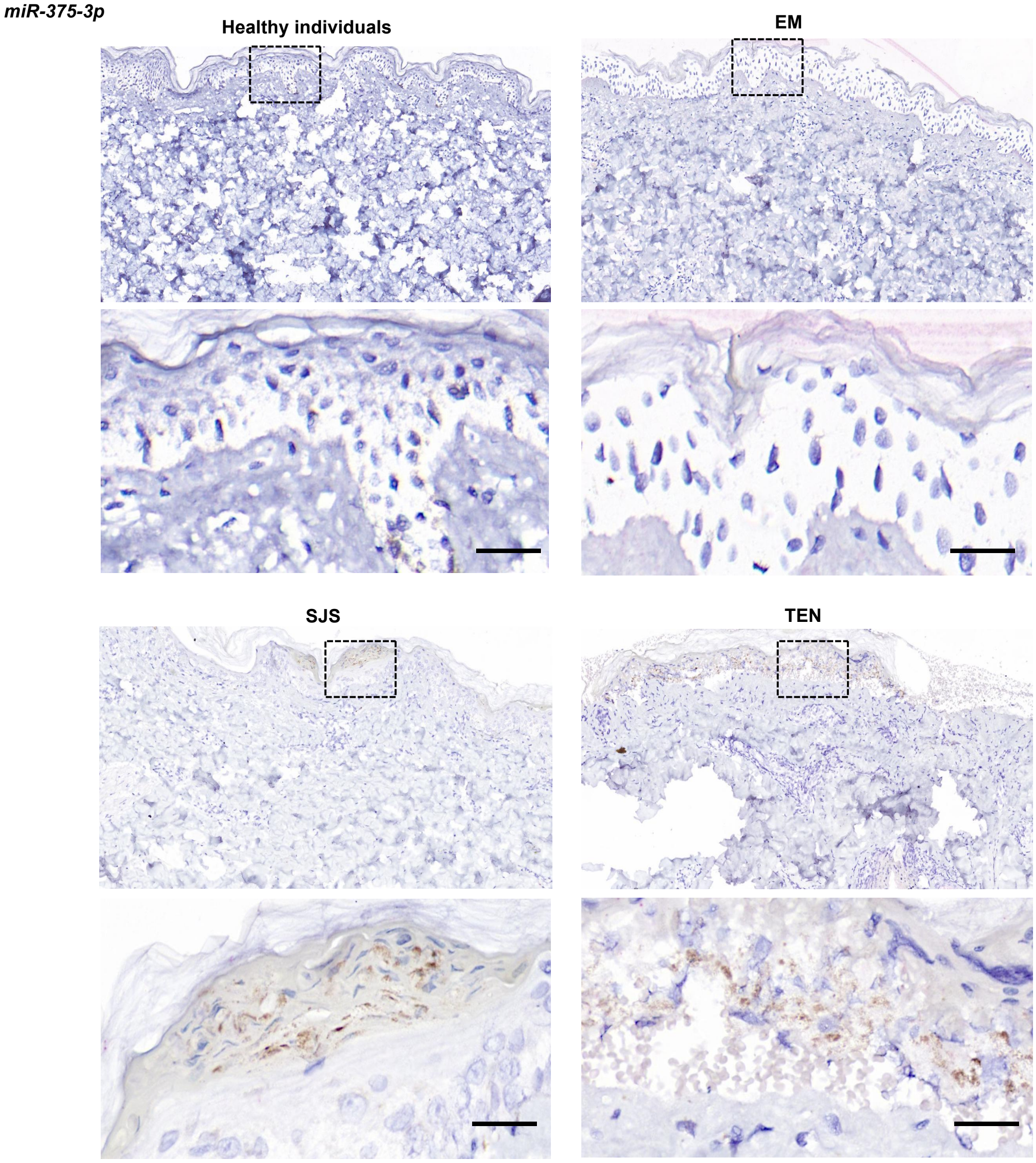
miR-375-3p
Healthy individuals
EM
SJS
TEN

Figure S10

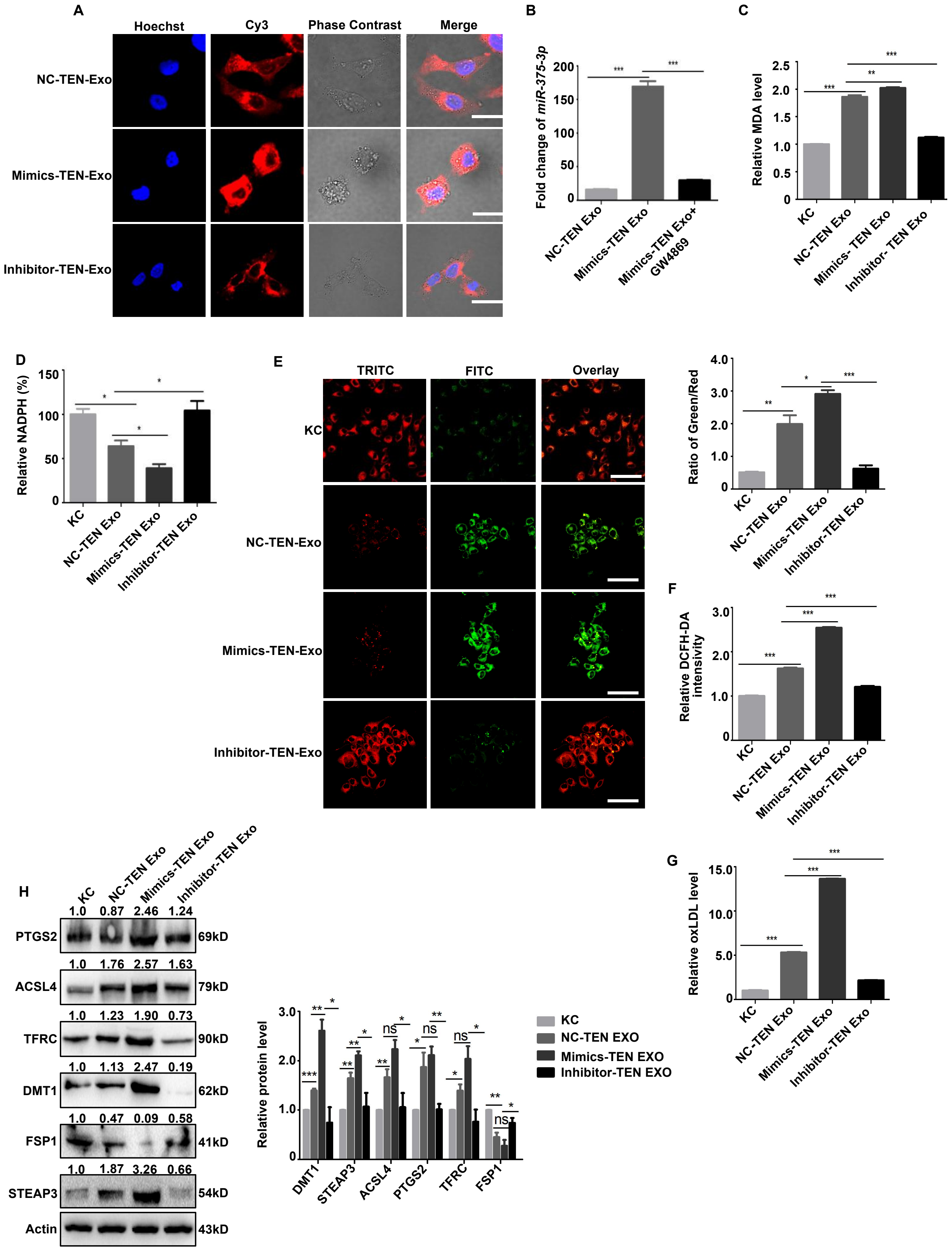
A
Hoechst
Cy3
Phase Contrast
Merge
NC-TEN-Exo
Mimics-TEN-Exo
Inhibitor-TEN-Exo
B
Fold change of miR-375-3p
NC-TEN Exo
Mimics-TEN Exo
Mimics-TEN Exo+ GW4869
C
Relative MDA level
KC
NC-TEN Exo
Mimics- TEN Exo
Inhibitor- TEN Exo
D
Relative NADPH (%)
KC
NC-TEN Exo
Mimics-TEN Exo
Inhibitor-TEN Exo
E
TRITC
FITC
Overlay
KC
NC-TEN-Exo
Mimics-TEN-Exo
Inhibitor-TEN-Exo
Ratio of Green/Red
F
Relative DCFH-DA intensivity
G
Relative oxLDL level
H
KC
NC-TEN Exo
Mimics-TEN Exo
Inhibitor-TEN Exo
1.0 0.87 2.46 1.24
PTGS2 69kD
1.0 1.76 2.57 1.63
ACSL4 79kD
1.0 1.23 1.90 0.73
TFRC 90kD
1.0 1.13 2.47 0.19
DMT1 62kD
1.0 0.47 0.09 0.58
FSP1 41kD
1.0 1.87 3.26 0.66
STEAP3 54kD
Actin 43kD
Relative protein level
DMT1
STEAP3
ACSL4
PTGS2
TFRC
FSP1
KC
NC-TEN EXO
Mimics-TEN EXO
Inhibitor-TEN EXO

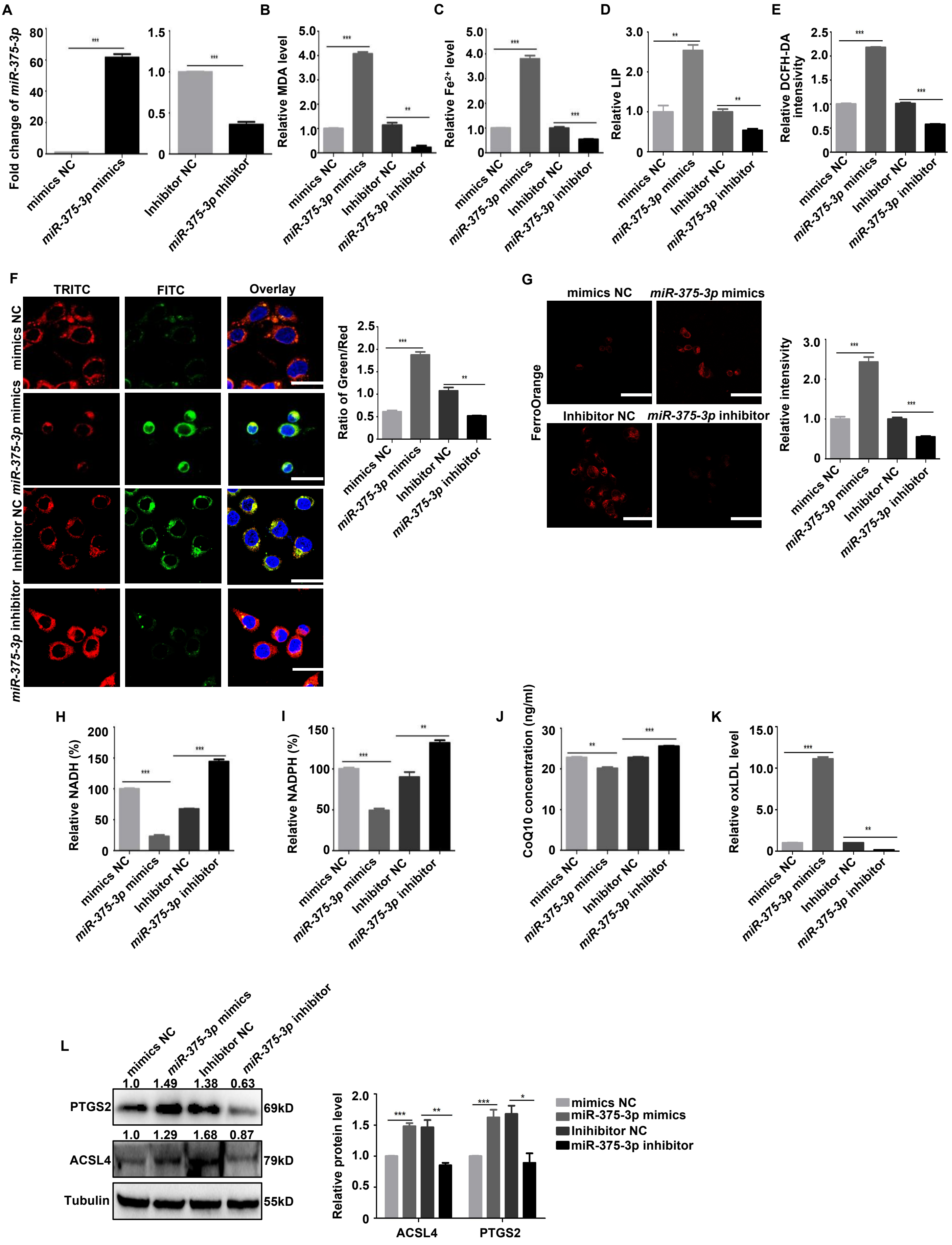
A
Fold change of miR-375-3p
mimics NC
miR-375-3p mimics
Inhibitor NC
miR-375-3p nhibitor
B
Relative MDA level
C
Relative Fe2+ level
D
Relative LIP
E
Relative DCFH-DA intensivity
F
TRITC
FITC
Overlay
mimics NC
miR-375-3p mimics
Inhibitor NC
miR-375-3p inhibitor
Ratio of Green/Red
G
FerroOrange
Relative intensity
H
Relative NADH (%)
I
Relative NADPH (%)
J
CoQ10 concentration (ng/ml)
K
Relative oxLDL level
L
PTGS2
1.0 1.49 1.38 0.63
69kD
ACSL4
1.0 1.29 1.68 0.87
79kD
Tubulin
55kD
Relative protein level
ACSL4
PTGS2
mimics NC
miR-375-3p mimics
Inihibitor NC
miR-375-3p inhibitor

A

GPX4
NM_002085-5utr
ATTGGTCGGCTGGACGAGGGGAGGAGCCGCTGGCTCCCAGCCCCGCCGCG
NM_002085-5utr(miR-375-3p)-mut
AGTGTGCTTAGTTCATCTGTTCGGAGCCGCTGGCTCCCAGCCCCGCCGCG

```
Target 5' A  UG  C               G    G 3'
            U  GU  GGCUGGACGAG  GGA
            G  CG  UCGGCUUGCUU  UUU
miRNA 3'A  UG  C               G       5'
```

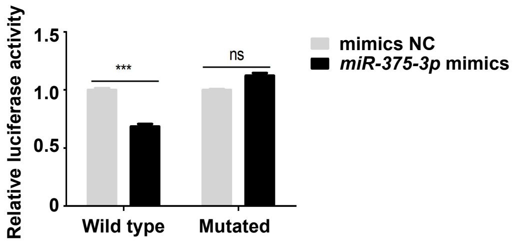

B

GCH1 WT-hsa-miR-375-3p
CCACGCACCTGAACGAGCACC
GCH1 MUT-hsa-miR-375-3p
CGTGCGACGACTTGCTCGTCC

```
Target    5' C        AC                   C   3'
             CACGC     CUGAACGAGCA
             GUGCG     GGCUUGCUUGU
miRNA     3' A        CUC                  UU 5'
```

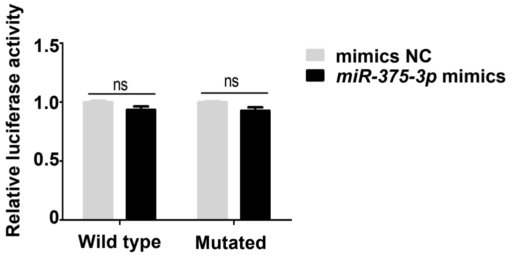

C

DHODH WT-hsa-miR-375-3p
TTCTGGTGTCGGTGCAGAGCAGGG
DHODH MUT-hsa-miR-375-3p
TAGTGCACAGCCTGCACTCGTCCG

```
target     5' U    UG            UGCA          G 3'
              UC    GU   GUCGG        GAGCAGG
              AG    CG   CGGCU        CUUGUUU
miRNA      3'     UG   CU         UG                5'
```

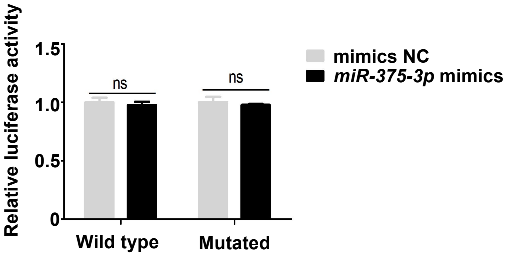

Figure S13

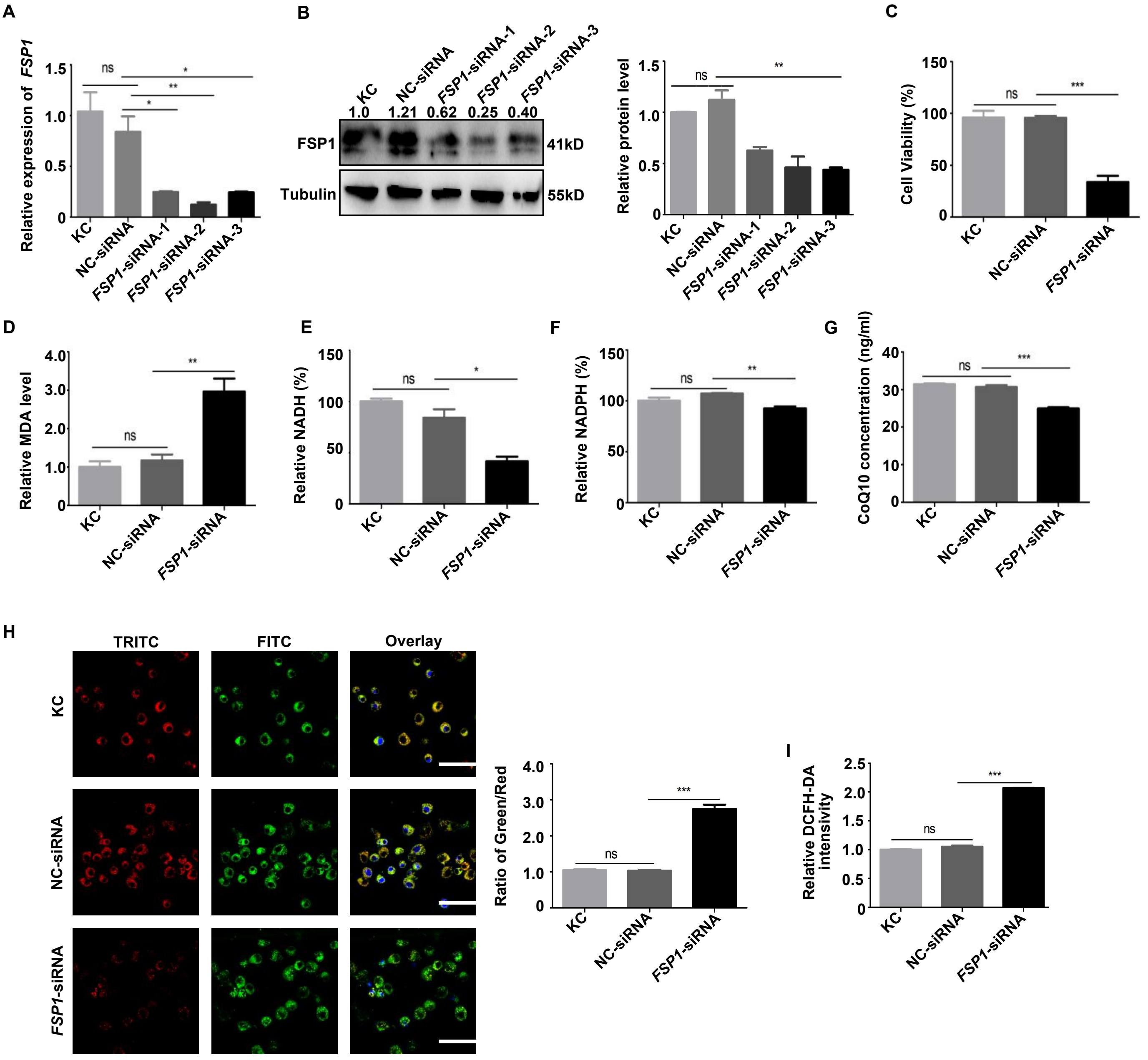

A
Relative expression of FSP1
1.5
1.0
0.5
0
ns
*
**
*
KC
NC-siRNA
FSP1-siRNA-1
FSP1-siRNA-2
FSP1-siRNA-3
B
KC
NC-siRNA
FSP1-siRNA-1
FSP1-siRNA-2
FSP1-siRNA-3
1.0 1.21 0.62 0.25 0.40
FSP1
41kD
Tubulin
55kD
Relative protein level
1.5
1.0
0.5
0
ns
**
C
Cell Viability (%)
150
100
50
0
ns
***
KC
NC-siRNA
FSP1-siRNA
D
Relative MDA level
4.0
3.0
2.0
1.0
0
ns
**
E
Relative NADH (%)
150
100
50
0
ns
*
F
Relative NADPH (%)
150
100
50
0
ns
**
G
CoQ10 concentration (ng/ml)
40
30
20
10
0
ns
***
H
TRITC
FITC
Overlay
KC
NC-siRNA
FSP1-siRNA
Ratio of Green/Red
4.0
3.0
2.0
1.0
0
ns
***
I
Relative DCFH-DA intensivity
2.5
2.0
1.5
1.0
0.5
0
ns
***

Figure S14

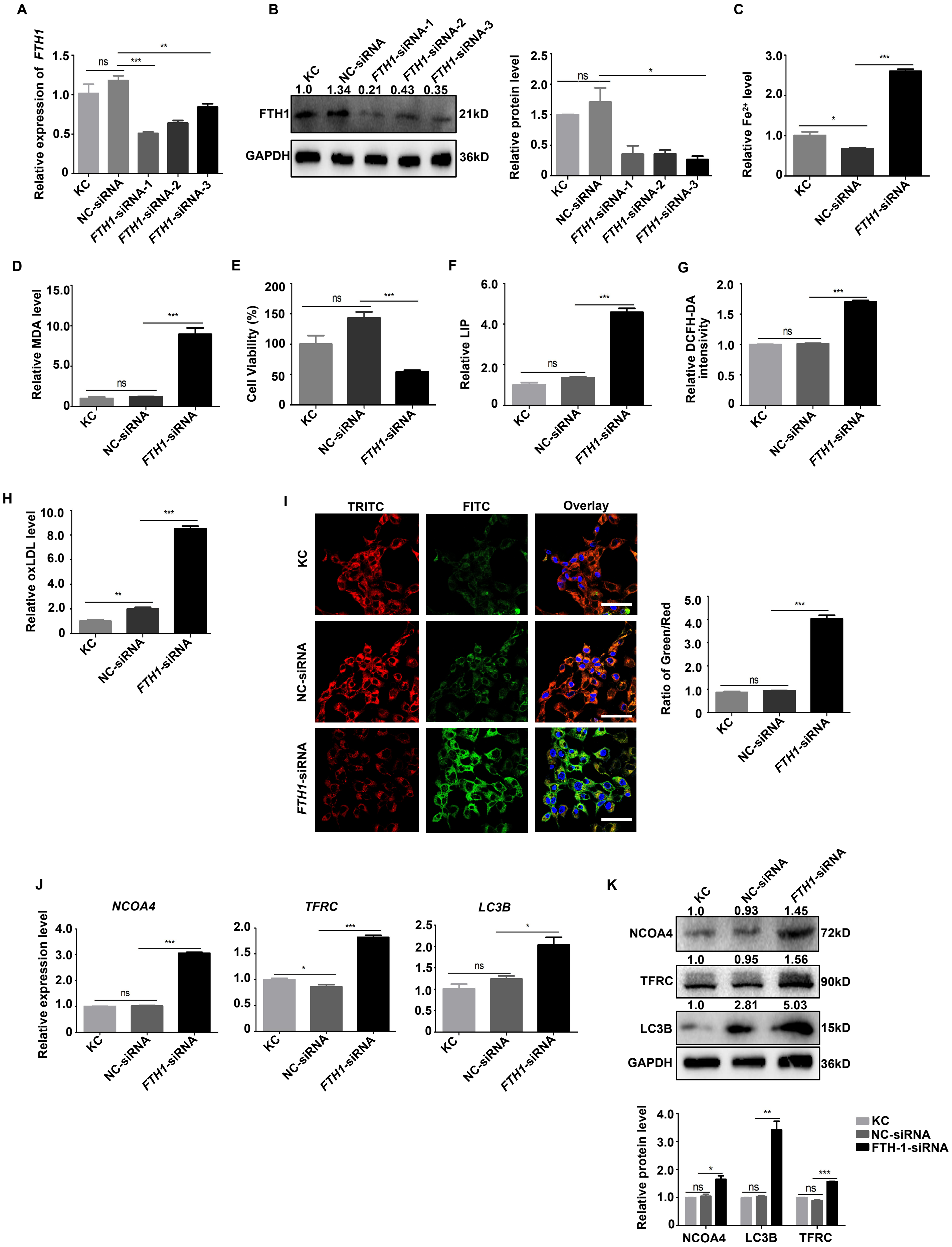
A
Relative expression of FTH1
KC
NC-siRNA
FTH1-siRNA-1
FTH1-siRNA-2
FTH1-siRNA-3
B
1.0 1.34 0.21 0.43 0.35
FTH1
21kD
GAPDH
36kD
Relative protein level
C
Relative Fe2+ level
FTH1-siRNA
D
Relative MDA level
E
Cell Viability (%)
F
Relative LIP
G
Relative DCFH-DA intensivity
H
Relative oxLDL level
I
TRITC
FITC
Overlay
Ratio of Green/Red
J
NCOA4
TFRC
LC3B
Relative expression level
K
1.0 0.93 1.45
NCOA4
72kD
1.0 0.95 1.56
TFRC
90kD
1.0 2.81 5.03
LC3B
15kD
GAPDH
36kD
Relative protein level
KC
NC-siRNA
FTH-1-siRNA

Figure S15

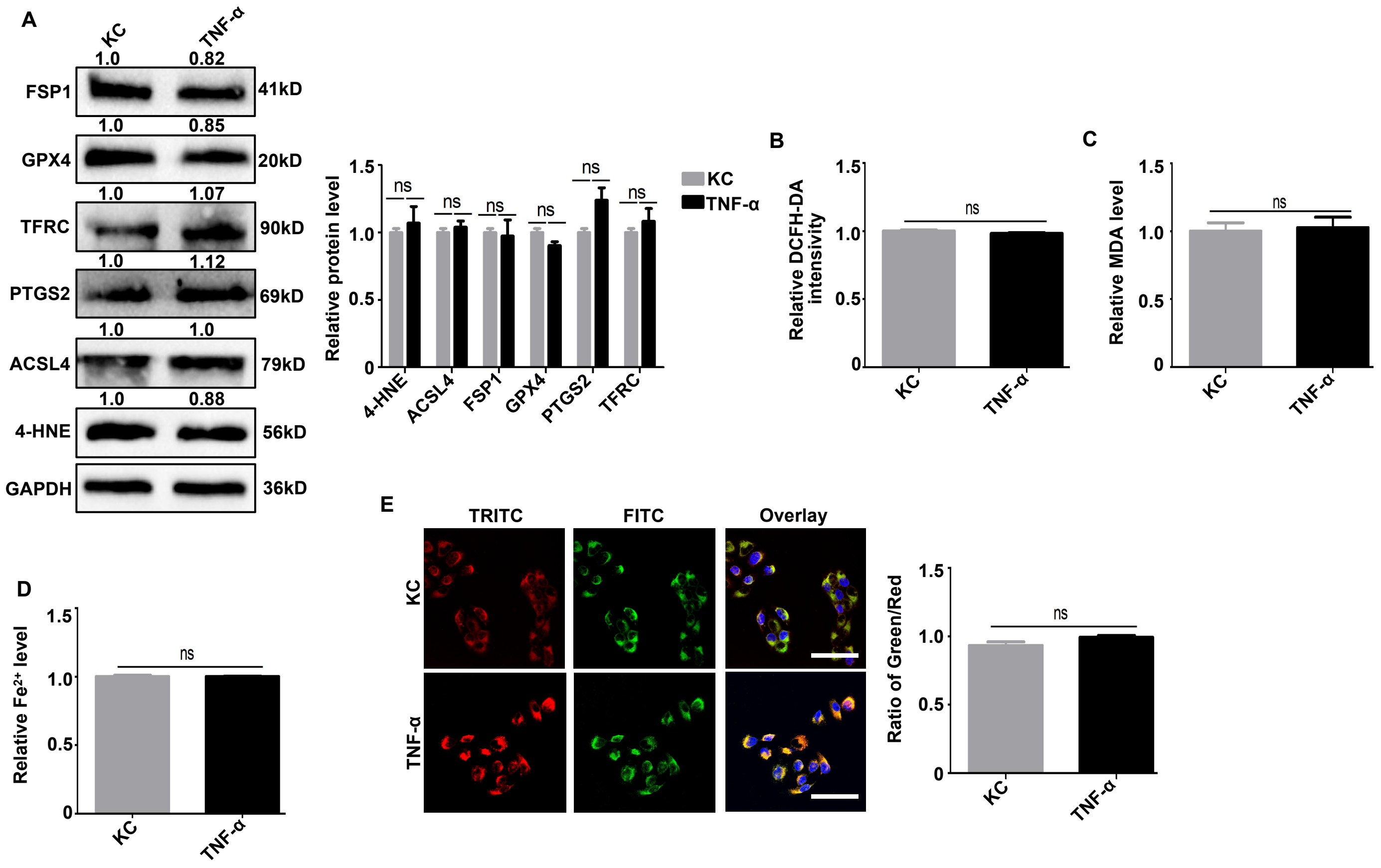
A
KC
TNF-α
1.0
0.82
FSP1
41kD
1.0
0.85
GPX4
20kD
1.0
1.07
TFRC
90kD
1.0
1.12
PTGS2
69kD
1.0
1.0
ACSL4
79kD
1.0
0.88
4-HNE
56kD
GAPDH
36kD
Relative protein level
1.5
1.0
0.5
0
ns
4-HNE
ACSL4
FSP1
GPX4
PTGS2
TFRC
KC
TNF-α
B
Relative DCFH-DA intensivity
ns
KC
TNF-α
C
Relative MDA level
ns
KC
TNF-α
D
Relative Fe²⁺ level
ns
KC
TNF-α
E
TRITC
FITC
Overlay
KC
TNF-α
Ratio of Green/Red
ns
KC
TNF-α

Figure S16

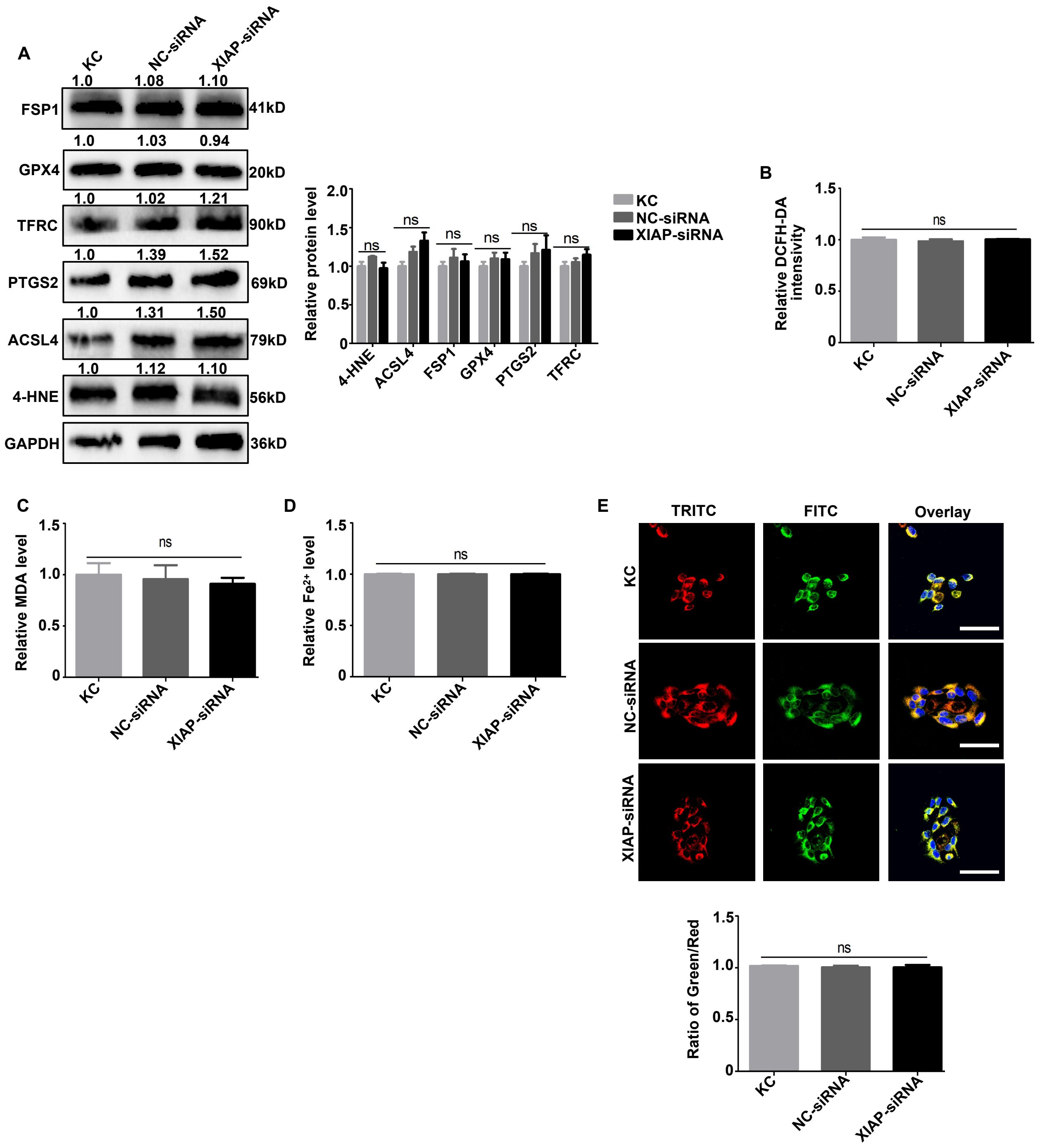
A
KC
NC-siRNA
XIAP-siRNA
FSP1
GPX4
TFRC
PTGS2
ACSL4
4-HNE
GAPDH
41kD
20kD
90kD
69kD
79kD
56kD
36kD
1.0 1.08 1.10
1.0 1.03 0.94
1.0 1.02 1.21
1.0 1.39 1.52
1.0 1.31 1.50
1.0 1.12 1.10
Relative protein level
ns
B
Relative DCFH-DA intensivity
C
Relative MDA level
D
Relative Fe2+ level
E
TRITC
FITC
Overlay
Ratio of Green/Red

Figure S17

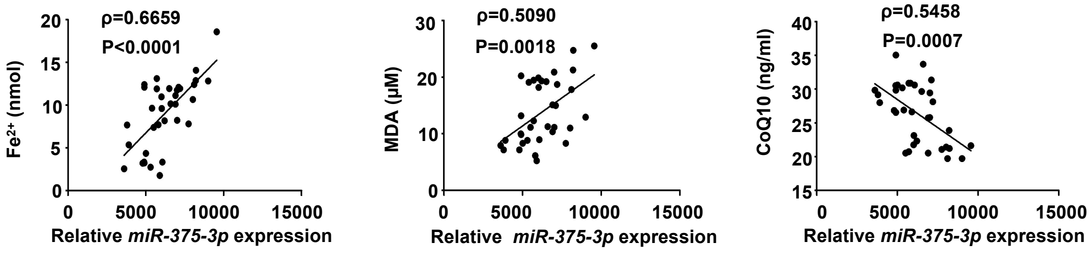
ρ=0.6659
P<0.0001
Fe²⁺ (nmol)
20
15
10
5
0
0
5000
10000
15000
Relative miR-375-3p expression
ρ=0.5090
P=0.0018
MDA (μM)
30
20
10
0
0
5000
10000
15000
Relative miR-375-3p expression
ρ=0.5458
P=0.0007
CoQ10 (ng/ml)
40
35
30
25
20
15
0
5000
10000
15000
Relative miR-375-3p expression
